# Widespread Evolution of Molecular Resistance to Snake Venom α-Neurotoxins in Vertebrates

**DOI:** 10.3390/toxins12100638

**Published:** 2020-10-02

**Authors:** Muzaffar A. Khan, Daniel Dashevsky, Harald Kerkkamp, Dušan Kordiš, Merijn A. G. de Bakker, Roel Wouters, Jory van Thiel, Bianca op den Brouw, Freek J. Vonk, R. Manjunatha Kini, Jawad Nazir, Bryan G. Fry, Michael K. Richardson

**Affiliations:** 1 Institute of Biology Leiden, Leiden University, Sylvius Laboratory, 2333 BE Leiden, The Netherlands; m.a.khan@biology.leidenuniv.nl (M.A.K.); h.m.i.j.kerkkamp@umail.leidenuniv.nl (H.K.); M.A.G.de.Bakker@biology.leidenuniv.nl (M.A.G.d.B.); woutersroel1@gmail.com (R.W.); joryvthiel@gmail.com (J.v.T.); freek.vonk@naturalis.nl (F.J.V.); 2 Toxin Evolution Lab, School of Biological Sciences, University of Queensland, St. Lucia, QLD 4072, Australia; danieldashevsky@gmail.com (D.D.); b.m.opdenbrouw@gmail.com (B.o.d.B.); 3 Australian National Insect Collection, Commonwealth Science & Industry Research Organisation, Acton, ACT 2601, Australia; 4 Department of Molecular and Biomedical Sciences, Josef Stefan Institute, 1000 Ljubljana, Slovenia; dusan.kordis@ijs.si; 5 Division of BioAnalytical Chemistry, Amsterdam Institute of Molecular and Life Sciences, Vrije Universiteit Amsterdam, De Boelelaan 1085, 1081 HV Amsterdam, The Netherlands; 6 Australian Venom Research Unit, Department of Pharmacology and Therapeutics, University of Melbourne, Melbourne, VIC 3010, Australia; 7 Naturalis Biodiversity Center Leiden, 2333 CR Leiden, The Netherlands; 8 Department of Biological Science, National University of Singapore, Singapore 117543, Singapore; dbskinim@nus.edu.sg; 9 Department of Pharmacology, Yong Loo Lin School of Medicine, National University of Singapore, Singapore 117600, Singapore; 10 Department of Microbiology, University of Veterinary and Animal Sciences, Lahore 54000, Pakistan; jawad.nazir@uvas.edu.pk; 11 Treidlia Biovet, 36/45 Powers Road, Seven Hills, NSW 2147, Australia

**Keywords:** evolutionary arms race, Elapidae, venom, resistance, nicotinic acetylcholine receptor (nAChR), CHRNA1, *N*-glycosylation

## Abstract

Venomous snakes are important subjects of study in evolution, ecology, and biomedicine. Many venomous snakes have alpha-neurotoxins (α-neurotoxins) in their venom. These toxins bind the alpha-1 nicotinic acetylcholine receptor (nAChR) at the neuromuscular junction, causing paralysis and asphyxia. Several venomous snakes and their predators have evolved resistance to α-neurotoxins. The resistance is conferred by steric hindrance from *N*-glycosylated asparagines at amino acids 187 or 189, by an arginine at position 187 that has been hypothesized to either electrostatically repulse positively charged neurotoxins or sterically interfere with α-neurotoxin binding, or proline replacements at positions 194 or 197 of the nAChR ligand-binding domain to inhibit α-neurotoxin binding through structural changes in the receptor. Here, we analyzed this domain in 148 vertebrate species, and assessed its amino acid sequences for resistance-associated mutations. Of these sequences, 89 were sequenced de novo. We find widespread convergent evolution of the *N*-glycosylation form of resistance in several taxa including venomous snakes and their lizard prey, but not in the snake-eating birds studied. We also document new lineages with the arginine form of inhibition. Using an in vivo assay in four species, we provide further evidence that *N*-glycosylation mutations reduce the toxicity of cobra venom. The nAChR is of crucial importance for normal neuromuscular function and is highly conserved throughout the vertebrates as a result. Our research shows that the evolution of α-neurotoxins in snakes may well have prompted arms races and mutations to this ancient receptor across a wide range of sympatric vertebrates. These findings underscore the inter-connectedness of the biosphere and the ripple effects that one adaption can have across global ecosystems.

## 1. Introduction

Venoms have evolved independently in multiple animal lineages [1,2]. When a venomous animal injects venom into a target animal (an event called ’envenomation’), venom toxins disrupt physiological processes, causing pain, incapacitation, or death. The fitness costs associated with envenomation can spur a co-evolutionary “arms race” between predator and prey [3,4,5,6]. Arms races can occur in a wide range of ecological interactions (e.g., host–parasite conflict), but the venoms we focus on in this study are primarily evolved as predatory adaptations [3,4,5,6].

The snake α-neurotoxins are members of the three-finger toxin (3FTx) family [7,8,9,10,11] and are major components of venoms from the families Elapidae and Colubridae [12,13,14,15,16,17]. Venomous snakes in these families are of considerable scientific interest, not least because they are responsible for numerous human fatalities [18], and because species possessing them—such as *Boiga irregularis* (the brown tree snake)—can cause ecological destruction as invasive species owing in part to the effectiveness of these toxins [19].

**Figure 1:**
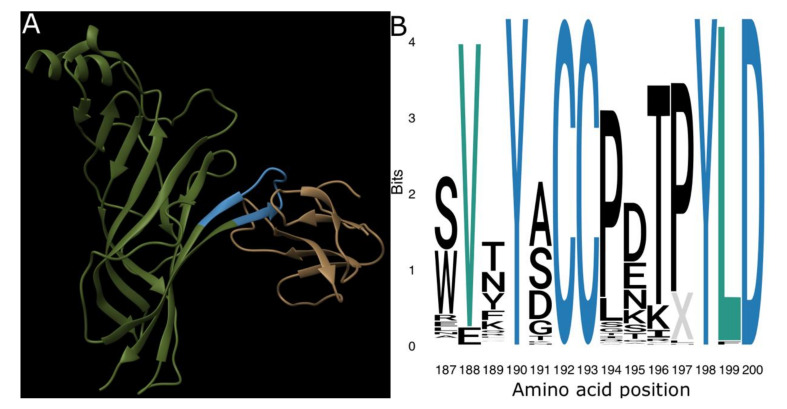
The ligand-binding domain of the nicotinic acetylcholine receptor (nAChR). (**A**) Ribbon model of α-bungarotoxin (brown) forming a complex with the ligand-binding domain (blue) on the extracellular domain of a single human α_1_-nAChR subunit (green). This structure is publicly available from the RCSB Protein Data Bank under the ID 6UWZ [20]. (**B**) Sequence logo showing the information value and amino acid content of the ligand-binding domain sequences in our dataset. Note the complete conservation of positions 190, 192, 193, 198, and 200 (blue) and strong conservation of positions 188 and 199 (teal). Logo was produced using the R ggseqlogo package [34].

In species susceptible to α-neurotoxins, the toxins bind to the nicotinic acetylcholine receptor (nAChR) primarily by binding to Loop C of the α1-subunit (referred to as the ligand-binding pocket, see Figure 1A), but also through interactions with the Cys Loop, Loop F, and neighboring delta and gamma subunits [20].

A number of species that are frequently envenomated by elapids, including predators and prey, or the snakes when they accidently bite themselves, have evolved resistance to these toxins [21,22,23,24,25,26,27]. The mechanism of resistance in these cases is modification of the ligand-binding domain of the nAChR. For example, several studies demonstrate that the binding of α-neurotoxins is disrupted by glycosylation of asparagine residues. The NXS/T motif (where X = any amino acid except proline) is an indicator of *N*-glycosylation [28,29,30]. Previous research has shown this motif to have evolved convergently in *Naja haje* (Egyptian cobra) and its predator, *Herpestes ichneumon* (Egyptian mongoose), but at different sites within the ligand-binding domain of the α-1 subunit. *N*-glycosylation at positions 187 (*H. ichneumon*) and 189 (*N. haje*) impedes binding via steric hindrance owing to the long carbohydrate chain preventing docking by the α-neurotoxins, rendering both species resistant [24,31,32]. Additionally, mutations to the proline subsite of the ligand-binding domain of the *H. ichneumon* nAChR (194L and 197H), and testing of an artificial variant of the *Mus musculus* (house mouse) sequence with a 194S mutation, result in decreased α-neurotoxin affinity [33]. This is presumably due to changes in the conformation of the binding pocket.

Other resistant animals, including *Mellivora capensis* (honey badger), *Erinaceus concolor* (southern white-breasted hedgehog) and *E. europaeus* (European hedgehog), and *Sus scrofa* (wild boar), have independently evolved amino acid substitutions from an aromatic residue to the positively charged arginine at position 187, which greatly reduces the affinity of α-bungarotoxin [32,35,36,37], possibly due to electrostatic repulsion of the positively charged neurotoxins. However, recent analysis of a new high-resolution structure of the toxin-receptor complex has suggested that this mutation may impart resistance due to steric hindrance instead [20].

Given the convergence of these mutations across a diversity of resistant taxa, and in light of the trophic importance of snake venoms, we posit that α-neurotoxin resistance may be present in more species than is currently documented. In this study, therefore, we assessed α1-nAChR sequences from 148 vertebrate species for evidence of resistance mutations within the ligand-binding domain, with a particular focus upon the *N*-glycosylated asparagine form of steric hindrance. This is a far larger species sample, with a greater taxonomic range, than has previously been analyzed. This allowed us to look for multiple independent instances of evolutionary change and gain new insight patterns of resistance evolution. We examined amino acid sites associated with α-neurotoxin resistance (Table 1). We used developmental toxicity assays to demonstrate that the mutations identified in our sequence analyses confered resistance against α-neurotoxins in vivo.

## 2. Results and Discussion

We sequenced de novo the nAChR ligand binding domain of 89 vertebrate species and obtained sequences for a further 59 species from The National Center for Biotechnology Information (NCBI; Supplementary Table S1). A preliminary search for sites under positive selection was made independently by one of us (D.K.) using a smaller subset of the main sequence collection (Methods, Supplementary File S1). Positively selected sites inferred under posterior probability (PP) > 0.95 were found (172, 177, 181, 187, 194, and 206). These positively-selected sites include sites 187 and 194, modifications of which are associated with toxin resistance.

**Figure 2:**
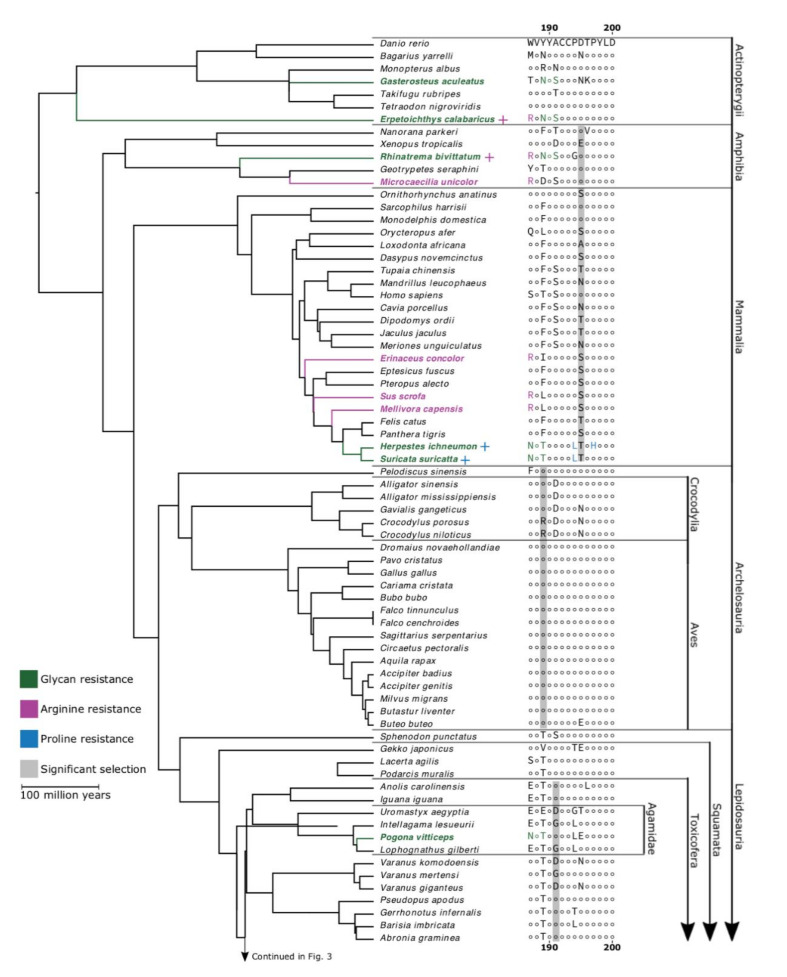
Sites of positive selection in α1-nAChR ligand-binding domain. Topology constructed from the consensus of TimeTree.org and taxon-specific phylogenies [43,44,45,46,47,48,49,50,51]. The most common amino acid sequence of the α1-nAChR ligand-binding is displayed for one species (*Danio rerio*) and differences from this sequence are displayed for all other species. Sites showing significant positive selection are highlighted in grey for the relevant clade. Green taxa and amino acids indicate resistance conferred through the glycosylated NXS/T motif, purple signifies the 187R mutation, and blue indicates resistance granted by proline subsite mutations. Scale bar indicates 100 million years of branch length. Continued in Figure 3.

In our analysis of the full dataset, we identified a number of highly-conserved sites, which was interesting given that our dataset covers a broad taxonomic scope and contains an over-representation of resistant species (Figure 1B). Conserved sites include the tyrosine residues at 190 and 198, which are known to interact directly with ligands, and the cysteine doublet at 192–193, which is crucial to the structure of the ligand-binding domain [39]. The conservation of these residues across such a diverse sampling of vertebrates suggests that they may be important to the physiological function of the endogenous neurotransmitter acetylcholine binding to the nAChR orthosteric site. By contrast, sites 187 and 189, sites of known α-neurotoxin resistance mutations, were far more variable than 194 and 197. Even though most of the observed variation at these sites came from mutations different from those that are known to produce resistance, we demonstrated that these α-neurotoxin resistance mutations are widespread among vertebrates. However, they were not found in any of the birds that we studied (Figure 2). The lack of resistance motifs for the snake-specialist predatory birds *Circaetus pectoralis* (black-chested snake eagle) and *Sagittarius serpentarius* (secretary bird) was particularly notable.

A number of species possess substitutions equivalent to those previously identified as conferring α-neurotoxin resistance, via steric hindrance imparted by *N*-glycosylation of an asparagine as they possess the well-documented signature NXS/T motif. The motif is present within two of the seven actinopterygian fish sampled: *Erpetoichthys calabaricus* (reedfish) and *Gasterosteus aculeatus* (three-spined stickleback). One of these, *E. calabaricus*, also possesses a 187R mutation, which is known to confer a different type of hindrance as seen in *Erinaceus* species, *M. capensis*, and *S. scrofa* [35,36,37,40,41]. Two species of South American caecilian, the tiny *Microcaecilia unicolor* (Cayenne caecilian) and *Rhinatrema bivittatum* (two-lined caecilian), possessed this 187R mutation, and *R. bivittatum* also displayed the 189–191NYS motif like that found in elapids. In contrast, the African caecilian species *Geotrypetes seraphini* (Gaboon caecilian) did not possess either resistance motif. *Suricata suricatta* (meerkat) and *Pogona vitticeps* (inland bearded dragon) both shared the 187–189NVT motif, which has been described in *H. ichneumon* [32,42]. Additionally, we find the 189–191NYS motif in all elapid snakes we examined, in addition to the *Naja* species in which it was originally characterised [24]. Variants of the NXS/T motif were found in other snakes that we sampled, occurring within subfamilies Viperinae (5/6 species), Natricinae (3/3 species), Colubrinae (4/13 species), and Dipsadinae (1/5 species).

**Figure 3:**
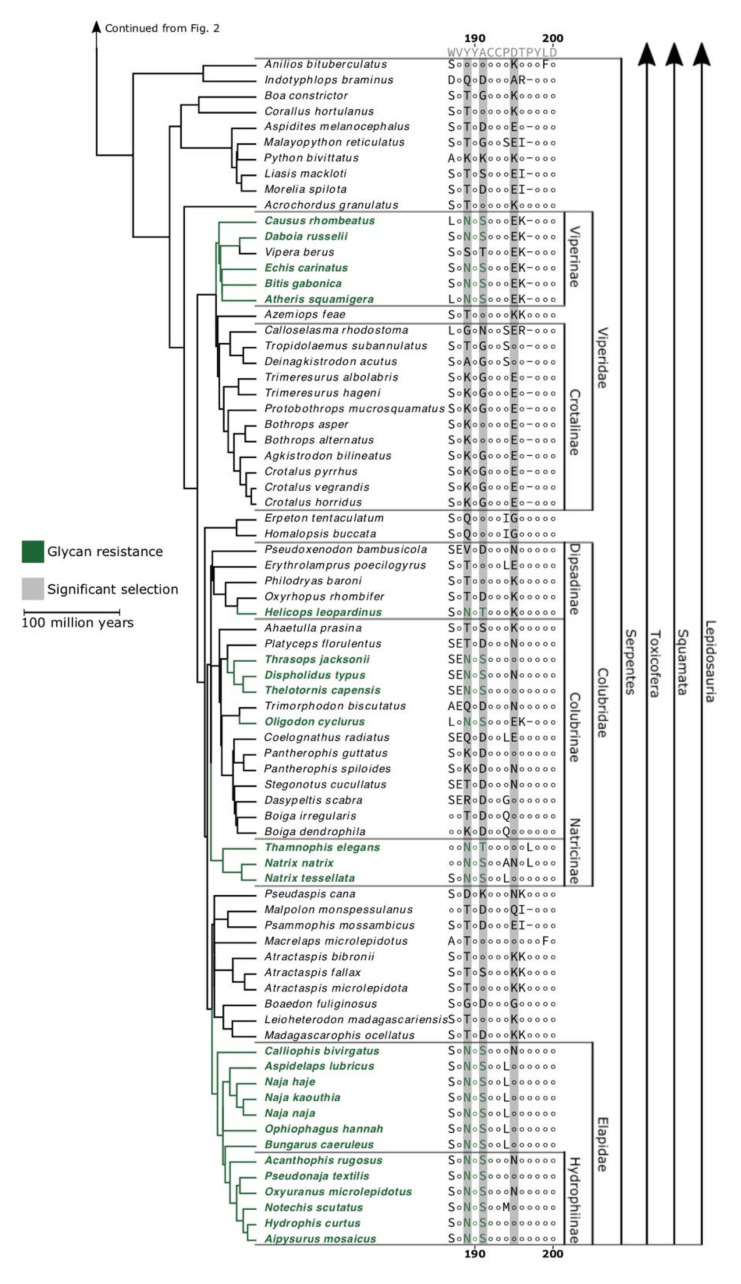
Continuation of Figure 2.

To support our conjecture that the *N*-glycosylated asparagine confers resistance in additional species, we demonstrated decreased mortality following exposure to α-neurotoxins in two species with these mutations, compared with two species without these mutations (Table 2). In this series of developmental toxicity assays, we used embryos of *G. aculeatus* and *P. vitticeps*, which possess mutations 187–189NVT and 189–191NYS, respectively. For comparison, we used the embryos of *Gallus gallus* (domestic chicken) and *Danio rerio* (zebrafish), both of which lack relevant mutations. The embryos were exposed to *Naja naja* venom in a concentration series to calculate the lethal dose or lethal concentration for 50% of embryos/larvae (LD_50_ and LC_50_, respectively). *G. aculeatus* tolerated approximately ten times more venom than *D. rerio* (LD_50_: 0.673 vs. 0.062 mg/mL), and *P. vitticeps* around five times that of *G. gallus* (LD_50_: 1.870 vs. 0.340 mg/mL).

None of the species in our dataset possessed the 197H mutation found in *H. ichneumon*. However, in our sequence analysis, we found that several species possess proline replacements at positions 194 and 197, identical to those that have been previously associated with resistance [33]. The 194L mutation is particularly widespread, and was found in the following: *Suricata suricatta* (meerkat); all three of the Australian agamids studied (*Intellagama lesueurii* (water dragon)*, Lophognathus gilberti* (Gilbert’s dragon)*,* and *P. vitticeps*); the anguimorph lizard *Barisia imbricata* (transvolcanic alligator lizard); the dipsadine snake *Erythrolamprus poecilogyrus* (yellow-bellied water snake); the natricine snake *Natrix tessellata* (dice snake); the colubrine snake *Coelognathus radiatus* (radiated ratsnake); and six out of the seven non-hydrophine elapid snakes sequenced (*Aspidelaps lubricus* (Cape coral cobra)*, Bungarus caeruleus* (blue krait)*, Naja haje* (Egyptian cobra)*, Naja kaouthia* (monocled cobra)*, Naja naja* (spectacled cobra)*,* and *Ophiophagus hannah* (king cobra)). We also found the 194S mutation in the pythonid snake *Malayopython reticuatus* (reticulated python) and in 3 of the 12 basal crotalines sequenced (*Calloselasma rhodostoma* (Malayan pit viper)*, Deinagkistrodon acutus* (sharp-nosed pit viper)*,* and *Tropidolaemus subannulatus* (Bornean keeled pit viper)), and a 194T mutation in the anguimorph lizard *Gerrhonotus infernalis* (Texas alligator lizard). The exact impact of these mutations is difficult to predict because the study that identified them suggested that there are complex patterns of interaction between mutations at positions 194 and 197, as well as between these mutations and those associated with steric hindrance resistance at positions 187 and 189 [33]. Thus, the results of species in this study identified as having replacements of prolines at positions 194 or 197 must be interpreted with caution. As mentioned above, even those specific substitutions that have been demonstrated to confer resistance in one taxon cannot confidently be stated to do so in others, especially mutations to amino acids that have never specifically been associated with resistance. For instance, α-neurotoxic venoms have been found to bind the ligand-binding domain sequences of both *C. radiatus*, which contains the 194L mutation, and *Gekko japonicus* (Schlegel’s Japanese gecko), which contains the 194T mutations, with higher affinity than they do to other species tested that did not have replacements of these prolines [55,56,57]. These findings underscore the fact that not all substitutions at these sites confer resistance (e.g., [57]) and that complex interactions, involving multiple amino acids, may be involved in conferring resistance. Thus, with the exception of the well-validated resistance conferred by *N*-glycosylation present at positions 187 or 189, other mutations cannot be attributed as conferring resistance until validated as such through functional testing.

A number of mutations apparent in our dataset have not previously been discussed in the context of α-neurotoxin resistance. The mutation to arginine at position 187 in *Erinaceus* species, *M. capensis* (honey badger), and *Sus scrofa* confers resistance that has been suggested to be due to electrostatic charge repulsion, but also due to steric hindrance. Regardless of the mechanism of action, it is unclear if this mutation would confer resistance if it occurs at a different location. The *N*-glycosylation mutation has been shown to be position independent, conferring resistance when occurring at position 187 as well as at 189. However, this mutation confers resistance owing to steric hindrance resulting as a consequence of the introduction of a very bulky branching glycan arrangement. In contrast, even if arginine conferred resistance at position 187 as a result of steric hindrance, as has been suggested, this may be due to a position-specific steric hindrance due to interactions with other amino acids in a very specific manner. Similarly, if arginine confers resistance as a result of the introduction of a positive charge, as has also been suggested, again this may be position specific. Therefore, while we have revealed species that have a mutation to an arginine at position 189, in the absence of functional testing, this cannot be inferred as conferring resistance. These species are *Monopterus albus* (swamp eel), the genus *Crocodylus* (2/2 species), and the colubrine snake *Dasypeltis scabra* (egg-eating snake). We hypothesise that it is unlikely that the 189R mutation confers resistance because steric hindrance from the 187R mutation is imposed as a result of very specific interactions between the toxin and the ligand-binding domain, and positions 187 and 189 interact with different parts of the toxin [20,57]. This is in contrast to the steric hindrance by *N*-glycosylation, which, because of the large glycan emerging from the asparagine, presents a much larger obstacle to binding, which can hinder the process from a wider variety of positions within the binding pocket of the nicotinic acetylcholine receptor.

We used signals-of-selection analyses to calculate the ratio of nonsynonymous (β) to synonymous (α) substitutions within the α1-nAChR nucleotide sequence. Non-synonymous mutations affect the biochemistry of the final gene product, while synonymous mutations do not. A scarcity of non-synonymous mutations suggests that deviations from the ancestral state may be deleterious, and thus selected against due to a fitness disadvantage resulting from less efficient binding by the endogenous neurotransmitter acetylcholine. Conversely, an overabundance of non-synonymous compared with synonymous mutations implies an adaptive process selecting for change or diversity, and this is a hallmark of evolutionary arms races. We thus used this ratio to infer negative selection (α > β), neutral evolution (α = β), and positive selection (α < β) of sites within the nAChR sequence.

**Figure 4:**
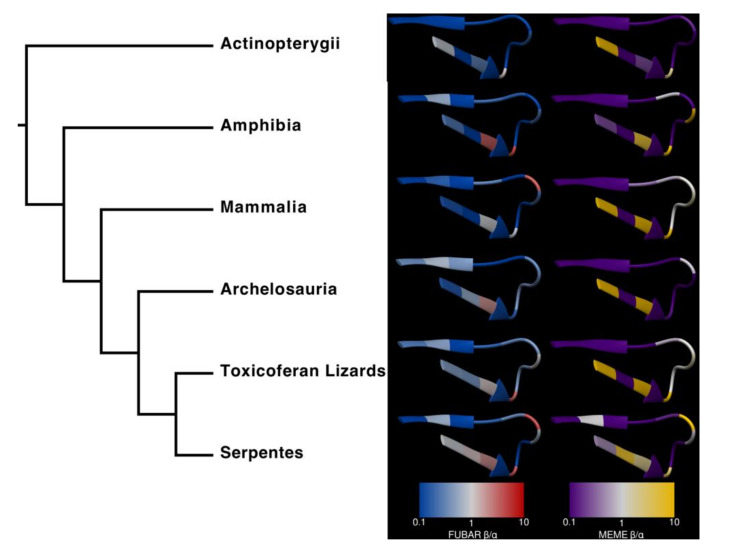
Amino acids in the α1-nAChR ligand-binding domains of snakes are subject to stronger and more pervasive positive selection than other taxa. The predicted surface of the ligand-binding domain (blue residues in Figure 1) is colored according to fast unconstrained Bayesian approximation (FUBAR) β/α and mixed effects model of evolution (MEME) weighted β/α, where red and yellow denote positive selection, while blue and purple represent negative selection. This structure is publicly available from the RCSB PDB under the ID 6UWZ; see also [20].

The analyses found several sites under significant positive selection (at the threshold of *p* < 0.1 for these conservative algorithms) within the ligand-binding domain in Figure 4. In the main analysis described here, we used mixed effects model of evolution (MEME) and fast unconstrained Bayesian approximation (FUBAR) to analyze the following clades: Actinopterygii, Mammalia, Archelosauria, Toxicoferan lizards, and Serpentes. MEME is designed to detect sites that have undergone episodic diversification, whereas FUBAR is built to detect sites with more pervasive positive selection throughout their evolutionary history. Because of these differences, we would expect MEME to determine a greater number of sites as significant than FUBAR, especially in lineages (such as Archelosauria) where relatively few taxa have the substitution, but those that do are closely related. While there was no significant positive selection within Actinopterygii, Amphibia (position 195: MEME *p* = 0.03, FUBAR *p* = 0.06), Mammalia (position 195: MEME *p* = 0.04, FUBAR *p* = 0.06), Archelosauria (position 189, MEME *p* = 0.01, FUBAR *p* = 0.18), and Toxicoferan lizards (position 191, MEME *p* = 0.06, FUBAR *p* = 0.07), all have one site under positive selection. In Serpentes, three positions, 189 (MEME *p* = 0.13, FUBAR *p* = 0.01), 191 (MEME *p* = 0.09, FUBAR *p* = 0.004), and 195 (MEME *p* = 0.001, FUBAR *p* = 0.001), were found to be significant by at least one of the analyses.

Both algorithms indicate that most remaining sites are subject to negative selection (α > β); sites that appear to be nearly neutral (α ≈ β) in some clades include those at positions 192, 193, 198, 199, and 200, which are strongly conserved across our species. This is an artefact arising from extraordinarily strong negative selection, which eliminates all or almost all mutations, including those that are non-synonymous, as discussed in [58].

**Actinopterygii**. To our knowledge, neither of the two fish species that were shown to possess the *N*-glycosylated form of neurotoxin resistance (*E. calabaricus* and *G. aculeatus*) have any evolutionary history as prey or predators of any α-neurotoxic snake species. Between this, the lack of any sites under significant positive selection (Figure 2), and the relatively strong negative selection across the ligand-binding domain (Figure 4), it is likely that these mutations are the result of an evolutionary process unrelated to our hypothesis. Nonetheless, we find evidence that the 189–191NYS motif in *G. aculeatus* does indeed reduce susceptibility to Indian cobra venom (Table 2). As discussed in the Lepidosauria section below, it appears that there is a fitness disadvantage to the *N*-glycosylation, with it being secondarily lost in lineages (e.g., *Vipera berus*) that have radiated out into areas outside the range of neurotoxic elapid snakes. Thus, the presence in two unrelated lineages of fish is intriguing and a fascinating area for future research. Sequencing of species related to those uncovered in this study as possessing the *N*-glycosylation mutation would be enlightening as to whether it is a trait that has recently evolved purely by chance in each species and has not yet to be subjected to purifying selection pressure. Conversely, if it is widely present in related species, that would suggest that it confers a benefit. As our functional testing showed that *G. aculeatus* is indeed resistant to neurotoxins, and putatively *E. calabaricus* as well, a hypothesis to test would be if this mutation confers resistance to anatoxin-a (also known as very fast death factor), a powerfully neurotoxic bicyclic amine alkaloidal cyanotoxin secreted by freshwater cyanobacteria that potently binds to nicotinic acetylcholine receptors [59].

**Amphibia**. Neither of the frog species included in our analyses possessed a resistance mutation, but two of the three caecilian species did. The resistant species are both South American which could be because of coral snakes (*Micrurus*), which are known to prey on caecilians, specifically including the tiny Cayenne caecilian (Martins and Oliveira 1998). From our phylogeny, it is impossible to be certain whether the 187R mutation is ancestral to all caecilians and further mutated to 187Y in the lineage, leading to *G. seraphini,* or whether it is a convergent mutation in *M. unicolor* and *R. bivittatum*. As these lineages predate the evolution of elapids, our hypothesis predicts the latter scenario, particularly as the two species possessing the well-characterized 187R resistance motif both occur sympatrically with *Micrurus* species, and thus would under significant predatory selection pressure. Further research into these enigmatic amphibians will be necessary to confirm or deny this prediction. A testable hypothesis is that caecilians from the Americas occurring sympatrically with fossorial elapids such as *Micrurus* would have widespread presence of resistance motifs, while conversely, caecilians not occurring sympatrically with fossorial elapids would lack resistance motifs.

**Mammalia**. We identified an additional species *S. suricatta* that was previously not known to possess resistance. The *S. suricatta* is closely related to *H. ichneumon* and has a similar foraging strategy, including venomous snakes in its diet, and anecdotally, *S. suricatta* is like *H. ichneumon* in being resistant to snake venom. This strongly suggests that the identical 187–189NVT sequence and the 194L mutations shared by these two taxa is a homologous trait that was present in their most recent common ancestor, which likely also preyed on venomous snakes. As with the amphibians, the consistent positive selection of site 195 across all mammals tested could be related to α-neurotoxin resistance, but this remains hypothetical until an actual effect or mechanism can be demonstrated.

**Archelosauria**. Ophiophagy (predation upon snakes) is common in birds of prey [60,61,62,63], and *Pavo cristatus* (Indian blue peafowl and *Cariama cristata* (red-legged seriema)) also sometimes feed on snakes [64]. Some species, such *Circaetus* sp (snake eagles) and *Sagittarius serpentarius* (secretary birds), are snake-specialist predators [65]. For these reasons, we predicted that resistance to α-neurotoxins would be present in birds. However, we found no resistance mutations within this clade. This widespread lack of resistance might help explain why the invasion of *B. irregularis* on Guam led to the eradication of so many local bird populations; *B. irregularis* venom is primarily composed of α-neurotoxins including the dimeric irditoxin, which binds especially well to the receptors of diapsids [66].

One possible explanation for this is that the predatory birds in this study already possess traits that potentially help them avoid envenomation. These include behavioural resistance traits (agility, high visual acuity, intelligence) and physical resistance traits (thick protective scalation on the legs and feathers on the body) [67,68]. Furthermore, birds typically rely on an ambush predation strategy, which likely reduces the risk of experiencing a defensive bite. Thus, the absence of resistance motifs within predatory birds that feed regularly on venomous snakes is suggestive of a fitness disadvantage for evolving neurotoxin resistance, whereby the advantage gained must outweigh the corresponding disadvantage. This suggestion is supported by secondary loss of resistance in viperid snakes that have radiated outside the range of neurotoxic predatory snakes (see Lepidosauria section below). Therefore, as predatory birds are not vulnerable to snakebite thanks to behavioural and mechanical forms for defense, they are not under selection pressure to evolve resistance and any random mutation conferring resistance would be under negative purifying selection pressure because of the impartation of a fitness disadvantage that is not offset by a proporationally greater fitness advantage.

As discussed above, the resistance in *Erinaceus* species, *Mellivora capensis*, and *Sus scrofa* by an arginine at position 187 is due to particular site-specific interaction, thus an arginine mutation at 189 does not automatically imply resistance. Thus, the mutation 189R revealed in the *Crocodylus* species *C. niloticus* and *C. porosus* cannot be attributed as conferring resistance in the absence of functional testing. The presence of 189R only in *Crocodylus,* but not in other crocodilians sequences, may be explained by the biogeographical history of the clade. *Crocodylus* diversified 13.6–8.3 million years ago (MYA) in Australasia after the split from all other Crocodylia 50 MYA, which diversified 70 MYA from Alligatoridae [69]. The diversification of the Elapidae began around 35 MYA in Asia [70]. This suggests that the speciation of the genus *Crocodylus* occurred in an environment occupied by Elapidae, while all other Crocodylia (alligators, gharials, and caiman) had diversified prior to that [69,70]. What remains unclear is the extent to which crocodiles interact with elapids when they share an environment. However, crocodiles are generalist predators, and given the common association between elapids and water bodies, it is plausible to posit that younger individuals may opportunistically predate upon elapids. This mutation in *Crocodylus* may explain why position 189 was found to be under significant positive selection in the Archelosauria, but again, it must be emphasized that there is no evidence that 189R confers resistance, and thus it cannot be inferred that *Crocodylus* species are resistant to snake neurotoxins. However, this is a rich area for future testing, as would sequencing of South American caimans that occur sympatrically with the aquatic elapid *Micrurus surinamensis* (aquatic coral snake).

**Lepidosauria**. *Sphenodon punctatus* (tuatara) possessed no resistance mutations. This ancient lineage diverged from the ancestor of Squamata around 220 MYA [71], roughly 185 million years before the origin of elapids, and has been isolated in a snake-free environment ever since. We found several resistance mutations within the lizards, most commonly proline substitutions, including all three Australian agamids (*I. lesueurii*, *L. gilberti*, and *P. vitticeps*). It is interesting to note that *P. vitticeps* is a relatively slow-moving species and possesses the key 187–189NVT motif, conferring steric hindrance owing to an *N*-glycosylated asparagine. In contrast, this motif is lacking in its swifter relative *L. gilberti*. This suggests that the evolution in the slow-moving lineage is due to its diminished ability to escape. Our developmental toxicity assays demonstrate that *P. vitticeps* is, in fact, resistant to α-neurotoxins. This is the first documented demonstration of α-neurotoxin resistance in a prey animal. The proline substitutions in the other lineages cannot be interpreted as imparting resistance; as noted above, the *C. radiatus* and *G. japonicus* have proline substitutions, but their receptor is nevertheless potently bound by α-neurotoxins [55,56,72].

Considering all the previously described resistance mutations, and the experimental evidence of resistance in snakes described in previous studies (*Eryx*, *Laticauda*, *Naja*, and *Natrix*) [24], our results suggest that the *N*-glycosylated asparagine form of α-neurotoxin resistance has evolved convergently at least six times within the snakes alone. The phylogenetic pattern provides evidence to suggest that these are independent origins of resistance rather than multiple losses. This is an extraordinary level of convergence of this very effective form of resistance.

We found mutations in the *N*-glycosylated asparagine form of α-neurotoxin resistance were particularly widespread in two of the major venomous snake families, Elapidae and Viperidae, but also occurred within Colubridae. Within the elapids, the 189–191NYS mutation is present in all 13 species examined, but was not in other closely related snake families. This suggests that it evolved once in the common ancestor of the elapids as a form of auto-resistance, paralleling the explosive diversification of α-neurotoxins within this family [73]. Within the viperids, only the Viperinae subfamily contains the *N*-glycosylated asparagine form of steric hindrance resistance (189–191NYS), which suggests that the selection pressure for resistance may postdate the divergence between these subfamilies. This leads us to posit that predation from ophiophagous elapids may have contributed to the evolution of this mutation in viperid snakes, given that the origin of elapids is thought to postdate the split between Viperinae and Crotalinae [46,51,70,74]. Interestingly, the European adder (*Vipera berus*), a viperine, is the only species examined here with a reversal of the *N*-glycosylated form of steric resistance (NXS/T mutation). This adder has a very broad distribution across northern Eurasia, however, it is found at relatively high latitudes and is not sympatric with any elapid [75]. This reversal may thus indicate that resistance mutations carry a fitness cost in a species that is no longer encountering α-neurotoxins. Such a scenario has been shown in several other cases of resistance to toxins [22,76]. Members of Colubridae are known to produce abundant α-neurotoxins within their venom, which could lead to the evolution of autoresistance [58,66,77,78]. Some of the taxa that possess resistance mutations are also sympatric with ophiophagous elapids, but in other cases such as Natricinae, it is less clear whether there was sufficient overlap between ancestral populations to lead to predator–prey coevolution, or whether these rear-fanged snake species produce high enough levels of α-neurotoxins to impart selection pressure for the evolution of auto-resistance.

While additional mechanisms of resistance may have evolved, such as mutations to the proline subsite, this will require future functional testing to validate. All of the non-hydrophine elapid snakes, except for *Calliophis bivirgatus* (Malaysian blue coral snake), share the 194L mutation. This suggests that it may have evolved in elapid snakes subsequent to the divergence of *Calliophis*, and was secondarily lost in the elapid snake lineage that colonized Australia, reverting back to the 194P ancestral state. Within the viperid snakes, the Crotalinae subfamily has the proline replacement (194S). It should be noted that the evidence linking this type mutation to α-neurotoxin resistance comes from a structure-function study of mammalian receptors, which demonstrated these changes resulted in significant resistance [33]. However, as the proline replacement mutations involve complex interplays between amino acids in the binding pocket, as opposed to the simple steric hindrance imposed by *N*-glycosylation of an asparagine, this mutation might not confer resistance in the context of the other differences between the mammalian and crotaline sequences. Thus, any level of resistance must be ascertained by functional testing before it can be attributed to these species.

Our examination of 148 nAChR sequences has revealed that numerous species, most of which were not previously known or suggested to possess α-neurotoxin resistance, contain mutations that have been shown to confer resistance or that, in the absence of pharmacological validation, have been suggested as strong candidates for conferring resistance. These various mutations are present within most of the classes in our dataset, with the unexpected exclusion of the birds. It was particularly noteable that the snake- specialist predatory birds *C. pectoralis* and *S. serpentarius* did not possess resistance motifs. There were also relatively few resistance-related mutations within the mammals. However, there were multiple convergent evolutions of the well-characterised *N*-glycosylation motif within the squamate reptiles—particularly the snakes. The greater sequence diversity (Figure 3), stronger positive selection, and weaker negative selection (Figure 4) across the ligand-binding domain in snakes compared with other lineages could result from the confluence of the three scenarios putatively selecting for the evolution of resistance: evolution of resistance to one’s own neurotoxins (autoresistance); evolution of resistance to the venom of α-neurotoxic prey; or evolution of resistance to the venom of an α-neurotoxic predators. Therefore, frequent exposure to α-neurotoxins could produce the widespread evolution of α-neurotoxin resistance via multiple independent selection pressures.

We also identified a number of sites under positive selection that were not previously associated with molecular mechanisms of venom resistance. A recent high-resolution model of an nAChR-α-bungarotoxin complex identified numerous sites of association between the receptor and toxin, including stabilising contact with subunit interfaces, interaction with loop F, and extensive contacts with loop C [20]. We thus postulate that mutations to additional sites of the nAChR could interfere with α-neurotoxin binding, and subsequently suggest that the mutations involved in this putative arms race are present in a wider array of positions than previously characterised. This is, therefore, a rich area for future functional testing, which may uncover entirely new forms of resistance to snake venom α-neurotoxins.

## 3. Conclusions

We conclude that the range of mechanisms along with the phylogenetic distribution of resistance to snake α-neurotoxin appears to be more extensive than previously appreciated. Our findings support the notion that the mutations we have identified may represent adaptive change in response to selective pressures exerted by α-neurotoxic snake venoms in an evolutionary arms race. Thus, we conclude that the evolutionary arms race between predator and prey appears to be a pervasive feature of the trophic interactions surrounding venomous snakes, which is shaping the molecular evolution of the nAChR in the vertebrates.

## 4. Materials and Methods

### 4.1. Ethics Statement

All animal experimental procedures were conducted in accordance with local and international regulations. The milking of snakes for venom is not considered an animal experiment in accordance with the Experiments on Animals Act (Wet op de dierproeven, 2014), the applicable legislation in the Netherlands, and its implementation the European guidelines (EU directive no. 2010/63/EU). The milking was executed in a licensed establishment for the breeding and use of experimental animals, and subject to internal regulations and guidelines; advice was taken from the Leiden University Ethics Committee to minimise suffering. In the case of snake embryos used for DNA extractions, no license is required by Council of Europe (1986), Directive 86/609/EEC. DNA samples from the NIH in Pakistan were harvested under local regulations of the National Institute of Health Islamabad, Pakistan. Blood samples from captive birds of prey in Pakistan were collected by J.N. and M.A.K., who are both qualified veterinary surgeons. The project was approved by the Ethics Committee of the University of Veterinary and Animal Sciences (UVAS), Lahore, Pakistan. No wild birds were caught specifically for this project, nor at our request or under our instructions. The material was collected before the ratification of the Nagoya protocol by Pakistan. No live animals in Australia were used; all samples studied were from existing tissue libraries (collected originally under University of Melbourne Animal Ethics approval number 03126.).

### 4.2. Tissue Samples

DNA was extracted from tissue samples preserved in 70% ethanol. The tissues were rinsed with 10% phosphate-buffered saline (PBS), then cut into small pieces, and transferred to DNA lysis buffer containing 10% sodium dodecyl sulfate (SDS) and 10 µL/mL Proteinase K overnight with gentle shaking at 55 °C digital heat block (VWR International, Amsterdam, The Netherlands). After the incubation, the buffer samples were centrifuged at high speed (20,238 rpm) for 15 min. The supernatant was mixed with isopropanol to precipitate the DNA and then centrifuged at high speed. The resultant pellet was washed with 70% ethanol, air dried, and dissolved in RNA/DNA free water at 65 °C for 45–60 min.

### 4.3. Amplification and Sequencing of the Ligand-Binding Domain of α-Neurotoxin nAChR

Primers specific for the ligand-binding domain of the nicotinic acetylcholine receptor (nAChR) were designed based on the alignment of reference sequences of the following snake species: *Naja haje* (Egyptian cobra), *Python bivittatus* (Burmese python), and *Ophiophagus hannah* (king cobra). For the birds, we used the sequences of *Haliaeetus leucocephalus* (bald eagle), *Falco peregrinus* (peregrine falcon), and *Gallus gallus* (domestic chicken), and for the lizards *Pogona vitticeps* (bearded dragon), we used *Anolis carolinensis* (green anole) and *Dopasia gracilis* (Asian glass lizard). Primer sequences are shown in Supplementary File S2 and the amplicon sequences in Supplementary File S3. Successively, an amplicon of 400 bp of the ligand-binding domain α-neurotoxin from the gene nAChR was amplified. PCR was performed in a volume of 25 µL mixture according to the instructions of the manufacturer (Qiagen, Inc., Carlsbad, CA, USA). We performed a touchdown PCR starting at an annealing temperature of 65 °C. As a quality check, the PCR products were electrophoresed for 30 min, and visualised on gel documentation apparatus (Westburg, Leusden, the Netherlands).

The amplified PCR products of nAChR for all snake species were Sanger-sequenced in both directions by BaseClear B.V., the Netherlands. All sequences were submitted to The National Center for Biotechnology Information (NCBI; https://www.ncbi.nlm.nih.gov/) and can be found under accession numbers: MN337792–MN337856, MT231203–MT231212, MT249118–MT249132, MT262918, MT262920, MT274611, and MT274612. Accession numbers of the NCBI reference sequences used are given in Supplementary Table S1 (Supplementary Materials).

### 4.4. Analysis of Site-Specific Selection

Nucleotide sequences of the ligand-binding domain from other species were downloaded from NCBI. The relevant accession numbers are given in Supplementary Table S1 (Supplementary Materials). The nucleotide sequences were translated into amino acids, manually aligned, and trimmed down to the 14 codons of the ligand-binding domain using AliView 1.18 (https://ormbunkar.se/aliview/) [79]. A phylogeny of all the species included in our dataset was compiled from a consensus generated by TimeTree.org and reconciled with taxon-specific phylogenies [43,44,45,46,47,48,49,50,51]. The data set was separated into five major clades: Actinopterygii, Mammalia, Archelosauria, toxicoferan lizards, and Serpentes. The tree data are given in Supplementary File S4. These were analysed using the FUBAR (fast unconstrained Bayesian approximation) and MEME (mixed effects model of evolution) programs implemented in HyPhy (hypothesis testing using phylogenies) 2.220150316beta [80,81,82].

### 4.5. Toxicity Assays Using Embryos

Functional assays of *Naja naja* (spectacled cobra) venom were performed on the following animals: *Gallus gallus* (chicken embryos, 5 d incubation); *Pogona vitticeps* (bearded dragon embryos, 5–7 d incubation); *Gasterosteus aculeatus* (three-spined stickleback larvae, 4 days post fertilization (4 dpf)); and *Danio rerio* (zebrafish larvae, 4 dpf). The bearded dragon and stickleback have a modified ligand-binding domain subunit of α-1nAChR consistent with α-toxin resistance, while the chicken and zebrafish do not (see main text).

#### 4.5.1. Preparation of Venom Stock Solution

*Naja naja* (spectacled cobra) venom was used in LD_50_ and LC_50_ functional assays. The venom was supplied by FJV. The venom was freeze-dried (lyophilised) and stored at −20 °C. For the experiments on the chicken and bearded dragon embryos, 7.7 mg/mL venom stock solution in sterile HBSS (Hanks’ balanced salt solution; Sigma Aldrich, H9269) was prepared. For *Gasterosteus aculeatus* and *Danio rerio*, the stock solution was also 7.7 mg/mL, but was prepared in egg water and tap water, respectively (that is, the swimming water for those two species). This yielded stock solutions with a venom concentration of 7.7 mg/mL. The stock solution was divided into 30 tubes in an amount of 100 µL per tube. These were stored at −80 °C.

#### 4.5.2. Embryo Set-Up

This LD_50_ assay was performed using embryos of *Gallus gallus* and *Pogona vitticeps*. The embryos were stored in a humidified incubator on stationary at 38 °C. *Pogona vitticeps* eggs were supplied from Reptielenhuis De Aarde, Breda, and Terrariumspeciaalzaak Kameleon, Tilburg, the Netherlands. *Pogona vitticeps* eggs were incubated in a humidified incubator at 28 °C. *Gasterosteus aculeatus* larvae were kindly provided Dr. Jörn Scharsack, Institute for Evolution and Biodiversity, Universität Münster, Germany, and were incubated at 17 °C. *Danio rerio* were obtained from the zebrafish facility of the Institute of Biology, Leiden University, and were incubated at 28 °C.

#### 4.5.3. LD_50_ Assay in *Gallus gallus* (Domestic Chicken) Embryos

*Gallus gallus* embryos of 5 d incubation were injected with 10 µL of venom solution. This solution was dropped onto the punctured vitelline membrane of the embryo, as described in [83]. A hole was made in the vitelline membrane with a tungsten needle. Four different venom concentrations were used: 1× (stock), 16×, 32×, and 64×, plus a control consisting of 10 µL of Hanks’ salt solution. The embryos were staged as described in [84]. The embryos were at stage 24 (Hamburger–Hamilton). Then, 10 µL venom was dripped onto the embryo with a Gilson P20 pipette through the previously-made hole. The egg was sealed afterwards with adhesive tape and returned to the incubator at 38 °C. The embryos were inspected 24 h after injection to see whether they were alive or dead.

#### 4.5.4. LD_50_ Assay in *Pogona vitticeps* (Inland Bearded Dragon) Embryos

There is no method described in the literature for LD_50_ assay on lizard embryos. Lizard eggs have a leathery, non-calcified shell and no air sac, and are thus extremely difficult to open without damage using the standard chicken embryo approach of ’windowing’. This is because the egg contents are very liable to herniate through the opened hole. We thus developed a new technique, which we describe here. The lizard embryos were staged as closely as possible to the Hamburger–Hamilton series. The position of the embryo was determined by candling, and a hole was made in the shell and shell membrane, just beyond the position of the embryo, using a sterilized syringe needle of gauge 26, L 1/2 inch. We then removed 30 to 50 µL of egg albumen using a sterile hypodermic 1 mL syringe. Then, 10 µL of venom solution was injected through the hole and under the shell membrane near the embryo using a Gilson P20 pipette. We did not, as we had in the chicken, puncture the vitelline membrane with a tungsten needle because of the danger of herniation of egg contents or damage to the embryo. However, it is at least possible that, in some cases, the vitelline membrane may have been ruptured by the hypodermic needle. This could not be determined, however, because of the lack of an air sac for windowing. The egg was sealed with an instant adhesive (Loctite 406; Henkel Adhesives, Düsseldorf, Germany) and incubated at 28 °C. The embryos were inspected 24 h after injection to determine whether the embryos were alive or dead.

#### 4.5.5. LC_50_ Assay on *Gasterosteus aculeatus* (Three-Spined Stickleback) and *Danio rerio* (Zebrafish) Developmental Stages

A geometric series was used, namely, 1× (stock), 2×, 4×, 8×, 16×, 32×, 64×, 128×, and 256×, plus a control consisting of 10 µL of vehicle (embryo medium). The diluted venom (60 µL) was introduced to each well of a 24-well tissue culture plate (VWR, 734-2325, VWR International, Radnor, Pennsylvania, USA) in which the single larvae were cultured, giving a total volume per well of 600 µL. One column of wells in the plate counted as controls. These control wells contained only 600 µL of embryo medium and a single larva.

The mortality of the developing *G. *aculeatus**) and *D. rerio* was recorded after 24 h. The following three criteria needed to be met for embryo to be scored as ’dead’: tissue opaque (milky-white) in appearance instead of transparent; heart not beating; and fish motionless (no locomotor activity). The LC_50_ values of *N. naja* venom were determined based on mortality scoring using Regression Probit analysis. This was achieved using the dose–response curve (drc) package in RStudio© (version 1.1.456; https://rstudio.com/). Details of the statistical analysis are given in Supplementary File S1.

## Figures and Tables

**Table 1:** Key sites and mutations that confer α-neurotoxin resistance.

Site	Mutation	Mechanism	Reference
187	NXS/T	Steric	[35]
	R	Steric	[38]
189	NXS/T	Steric	[24]
194	L	Proline	[33]
	S	Proline	[33]
197	H	Proline	[33]

**Table 2:** Pharmacological assays of cobra venom toxicity. Probit analysis was used to calculate the LD_50_ or LC_50._ For full details of the statistical analysis, see Supplementary File S1 and [52,53,54]. Key: LD_50_, LC_50_, lethal dose or lethal concetration, respectively, for 50% of embryos/larvae.

Concentration of *Naja naja* Venom (mg/mL)											
	0.00	0.03	0.06	0.12	0.24	0.48	0.945	1.89	3.78	7.7	LD_50_ or LC_50_ mg/mL
*Pogona vitticeps* (inland bearded dragon)											1.87
alive	5	-	-	5	5	5	-	-	-	0	
dead	0	-	-	0	0	0	-	-	-	5	
*Gallus gallus* (domestic chicken)											0.340
alive	5	-	-	5	5	0	-	-	-	0	
dead	0	-	-	0	0	5	-	-	-	5	
*Gasterosteus aculeatus* (three-spined stickleback)											0.673
alive	8	8	8	8	8	8	0	0	0	0	
dead	0	0	0	0	0	0	8	8	8	8	
*Danio rerio* (zebrafish)											0.062
alive	8	8	5	0	0	0	0	0	0	0	
dead	0	0	3	8	8	8	8	8	8	8	

## Supplementary Materials

The following are available online at https://www.mdpi.com/2072-6651/12/10/638/s1, Supplementary Table S1: Sequences included in this study, with accession numbers and species names; Supplementary File S1: Estimating LD_50_ values; Supplementary File S2: Primers used in the current study for amplification of Chrna1 (cholinergic receptor nicotinic alpha 1 subunit) ligand binding site; Supplementary File S3: Amplicons. Supplementary File S4: Tree file; Supplementary Note S1: Preliminary (independent) analysis of a subset of the data.
