# Supplementary material for: Widespread Evolution of Molecular Resistance to Snake Venom α-Neurotoxins in Vertebrates
Source: Toxins (Basel). 2020 Oct 2;12(10):638. doi: 10.3390/toxins12100638 (PMC7601176; doi:10.3390/toxins12100638)
Supplement: Supplementary file 1 [file toxins-12-00638-s001.zip › Supplementary Table S1 Sequences included in this study, with accession numbers and species names.docx]

**Supplementary Table S1**. Sequences included in this study, with accession numbers and species names. Species in the same order as in Figure 2. Key: ‘Source’ indicates the origin of the sequence or the DNA sample. In the case of the sequences determined by us de novo in this study, the authors of this paper who sourced the DNA samples are listed in the column headed ‘Author’ using the following abbreviations: BGF, Bryan G. Fry; FJV, Freek J. Vonk; HMIK, Harald M.I. Kerkkamp; JvT, Jory van Thiel; MAGdB, Merijn A.G. de Bakker; MAK, Muzaffar A. Khan; RMW, Roel M. Wouters. The remaining sequences were obtained from NCBI (NCBI, National Center for Biotechnology Information, Bethesda, Maryland, United States).

| **NCBI Accession No.** | **Scientific Name** | **Common Name** | **Source** | **Author** |
| --- | --- | --- | --- | --- |
| NM_131445.1 | *Danio rerio* | Zebrafish | GenBank, NCBI |  |
| VCAZ01000208.1 | *Bagarius yarrelli* | Giant devil catfish | GenBank, NCBI |  |
| XM_020601562.1 | *Monopterus albus* | Swamp eel | GenBank, NCBI |  |
| VDFK01000470.1 | *Gasterosteus aculeatus* | Three-spined stickleback | GenBank, NCBI |  |
| AY295875.1 | *Takifugu rubripes* | Japanese puffer | GenBank, NCBI |  |
| CAAE01015010.1 | *Tetraodon nigroviridis* | Spotted green pufferfish | GenBank, NCBI |  |
| XM_028808143.1 | *Erpetoichthys calabaricus* | Reedfish | GenBank, NCBI |  |
| XM_018572442.1 | *Nanorana parkeri* | High Himalaya frog | GenBank, NCBI |  |
| XM_002934601.5 | *Xenopus tropicalis* | African clawed frog | GenBank, NCBI |  |
| XM_029605825.1 | *Rhinatrema bivittatum* | Two-lined caecilian | GenBank, NCBI |  |
| XM_033946945.1 | *Geotrypetes seraphini* | Gaboon caecilian | GenBank, NCBI |  |
| XM_030209957.1 | *Microcaecilia unicolor* | Tiny Cayenne caecilian | GenBank, NCBI |  |
| XM_001514832.4 | *Ornithorhynchus anatinus* | Platypus | GenBank, NCBI |  |
| XM_003763981.2 | *Sarcophilus harrisii* | Tasmanian devil | GenBank, NCBI |  |
| XM_001376625.4 | *Monodelphis domestica* | Gray short-tailed opossum | GenBank, NCBI |  |
| XM_007940110.1 | *Orycteropus afer* | Aardvark | GenBank, NCBI |  |
| XM_023542827.1 | *Loxodonta africana* | African savanna elephant | GenBank, NCBI |  |
| XM_004476894.2 | *Dasypus novemcinctus* | Nine-banded armadillo | GenBank, NCBI |  |
| XM_006151116.1 | *Tupaia chinensis* | Chinese tree shrew | GenBank, NCBI |  |
| XM_011991880.1 | *Mandrillus leucophaeus* | Drill | GenBank, NCBI |  |
| NM_001039523.3 | *Homo sapiens* | Human | GenBank, NCBI |  |
| XM_003478585.3 | *Cavia porcellus* | Domestic guinea pig | GenBank, NCBI |  |
| XM_013028276.1 | *Dipodomys ordii* | Ord's kangaroo rat | GenBank, NCBI |  |
| XM_004660327.1 | *Jaculus jaculus* | Lesser Egyptian jerboa | GenBank, NCBI |  |
| XM_021649964.1 | *Meriones unguiculatus* | Mongolian gerbil | GenBank, NCBI |  |
| U17016.1 | *Erinaceus concolor* | Southern white-breasted hedgehog | GenBank, NCBI |  |
| XM_008138537.2 | *Eptesicus fuscus* | Big brown bat | GenBank, NCBI |  |
| XM_006921218.1 | *Pteropus alecto* | Black flying fox | GenBank, NCBI |  |
| XM_021075437.1 | *Sus scrofa* | Pig | GenBank, NCBI |  |
| KR477832.1 | *Mellivora capensis* | Honey badger | GenBank, NCBI |  |
| XM_003990883.5 | *Felis catus* | Domestic cat | GenBank, NCBI |  |
| XM_007074557.2 | *Panthera tigris* | Tiger | GenBank, NCBI |  |
| M93639.1 | *Herpestes ichneumon* | Egyptian mongoose | GenBank, NCBI |  |
| XM_029932975.1 | *Suricata suricatta* | Meerkat | GenBank, NCBI |  |
| XM_006119477.3 | *Pelodiscus sinensis* | Chinese soft-shelled turtle | GenBank, NCBI |  |
| XM_006020803.2 | *Alligator sinensis* | Chinese alligator | GenBank, NCBI |  |
| XM_006267516.3 | *Alligator mississippiensis* | American alligator | GenBank, NCBI |  |
| XM_019522952.1 | *Gavialis gangeticus* | Gharial | GenBank, NCBI |  |
| XM_019554696.1 | *Crocodylus porosus* | Saltwater crocodile | GenBank, NCBI |  |
| MT249132 | *Crocodylus niloticus* | Nile crocodile | La Ferme aux Crocodiles, Pierrelatte, France | MAGdB |
| XM_026092832.1 | *Dromaius novaehollandiae* | Emu | GenBank, NCBI |  |
| MT231212 | *Pavo cristatus* | Indian peafowl | Pet trade | MAK |
| MT274612 | *Gallus gallus* | Chicken | Pet trade | MAK |
| MT262918 | *Cariama cristata* | Red-legged seriema | Gaia Zoo, Kerkrade, Netherlands | RMW |
| MT231210 | *Bubo bubo* | Eurasian eagle-owl | Pet trade | MAK |
| MT231209 | *Falco tinnunculus* | Common kestrel | Pet trade | MAK |
| MT231206 | *Falco cenchroides* | Nankeen Kestrel | Pet trade | MAK |
| VWYJ01026266.1 | *Sagittarius serpentarius* | Secretary bird | GenBank, NCBI |  |
| VZZV01000171.1 | *Circaetus pectoralis* | Black-chested snake eagle | GenBank, NCBI |  |
| MT231205 | *Aquila rapax* | African tawny-eagle | Pet trade | MAK |
| MT231204 | *Accipiter badius* | Shikra | Pet trade | MAK |
| MT231203 | *Accipiter gentilis* | Northern goshawk | Pet trade | MAK |
| MT231207 | *Milvus migrans* | Black kite | Pet trade | MAK |
| MT231208 | *Butastur liventer* | Rufous-winged buzzard | Pet trade | MAK |
| MT231211 | *Buteo buteo* | Common buzzard | Pet trade | MAK |
| QEPC01008869.1 | *Sphenodon punctatus* | Tuatara | GenBank, NCBI |  |
| XM_015426640.1 | *Gekko japonicus* | Schlegel's Japanese gecko | GenBank, NCBI |  |
| XM_033167788.1 | *Lacerta agilis* | Sand lizard | GenBank, NCBI |  |
| XM_028749253.1 | *Podarcis muralis* | Common wall lizard | GenBank, NCBI |  |
| XM_003226425.3 | *Anolis carolinensis* | Green anole | GenBank, NCBI |  |
| MT249123 | *Iguana iguana* | Common green iguana | Pet trade | BGF |
| MT249130 | *Uromastyx aegyptia* | Egyptian spiny-tailed lizard | Pet trade | BGF |
| MT249127 | *Intellagama lesueurii,* | Eastern water dragon | Pet trade | BGF |
| MT249122 | *Pogona vitticeps* | Bearded dragon | Reptielenhuis de Aarde, Breda, the Netherlands | MAGdB |
| MT249128 | *Lophognathus gilberti* | Gilbert's lashtail | Pet trade | BGF |
| MT249129 | *Varanus komodoensis* | Komodo dragon | Pet trade | BGF |
| MT249118 | *Varanus mertensi* | Mertens' water monitor | Pet trade | BGF |
| MT249131 | *Varanus giganteus* | Perentie | Pet trade | BGF |
| MT249121 | *Pseudopus apodus* | Scheltopusik, Pallas's glass lizard | Terrariumspeciaalzaak Kameleon, Tilburg, the Netherlands | JvT & RMW |
| MT249120 | *Gerrhonotus infernalis* | Texas alligator lizard | Pet trade | BGF |
| MT249126 | *Barisia imbricata* | Transvolcanic alligator lizard | Pet trade | BGF |
| MT249119 | *Abronia graminea* | Mexican alligator lizard | Pet trade | BGF |
| MN337817 | *Anilios bituberculatus* | Prong-snouted blind snake | Pet trade | FJV |
| MT274611 | *Indotyphlops braminus* | Brahminy blind snake | Pet trade | BGF |
| MN337822 | *Boa constrictor* | Common boa | Pet trade | BGF |
| MN337841 | *Corallus hortulanus* | Garden tree boa | Pet trade | BGF |
| MN337819 | *Aspidites melanocephalus* | Black-headed python | Pet trade | BGF |
| MN337856 | *Malayopython reticuatus* | Reticulated python | Pet trade | BGF |
| XM_007444717 | *Python bivittatus* | Burmese python | GenBank, NCBI |  |
| MN337828 | *Liasis mackloti* | Macklot's water python | Pet trade | BGF |
| MN337853 | *Morelia spilota* | Carpet python | Pet trade | BGF |
| MN337818 | *Acrochordus granulatus* | Banded file snake | Pet trade | BGF |
| MN337801 | *Causus rhombeatus* | Rhombic night adder | Pet trade | FJV |
| MN337797 | *Daboia russelii* | Russell's viper | National Institute of Health (NIH) Islamabad, Pakistan | MAK |
| GCA_000800605.1 | *Vipera berus* | European adder | GenBank, NCBI |  |
| MN337798 | *Echis carinatus* | Saw-scaled viper | National Institute of Health (NIH) Islamabad, Pakistan | MAK |
| MN337800 | *Bitis gabonica* | Gaboon viper | Pet trade | BGF |
| MN337799 | *Atheris squamigera* | African bush viper | Gifttierhause Eimsheim, Germany. | JvT & RMW |
| MN337820 | *Azemiops feae* | Fea's viper | Pet trade | BGF |
| MN337855 | *Calloselasma rhodostoma* | Malayan pit viper | Pet trade | FJV |
| MN337851 | *Tropidolaemus subannulatus* | North Philippine temple pitviper | Pet trade | BGF |
| MN337844 | *Deinagkistrodon acutus* | Chinese moccasin | Gifttierhause Eimsheim, Germany. | JvT & RMW |
| MN337836 | *Trimeresurus albolabris* | White-lipped tree viper | Pet trade | BGF |
| MN337837 | *Trimeresurus hageni* | Indonesian pit viper | Pet trade | FJV |
| XM_015815894.1 | *Protobothrops mucrosquamatus* | Brown-spotted pit viper | GenBank, NCBI |  |
| MN337854 | *Bothrops asper* | Fer-de-lance | Gifttierhause Eimsheim, Germany. | JvT & RMW |
| MT262920 | *Bothrops alternatus* | Urutu | Gifttierhause Eimsheim, Germany. | JvT & RMW |
| MN337838 | *Agkistrodon bilineatus* | Cantil viper | Gifttierhause Eimsheim, Germany. | JvT & RMW |
| JPMF01213521.1 | *Crotalus pyrrhus* | Speckled rattlesnake | GenBank, NCBI |  |
| LVCR01006207.1 | *Crotalus horridus* | Timber rattlesnake | GenBank, NCBI |  |
| MN337852 | *Crotalus vegrandis* | Uracoan Rattlesnake | Pet trade | JvT & RMW |
| MN337824 | *Erpeton tentaculatum* | Tentacle snake | Pet trade | BGF |
| MN337825 | *Homalopsis buccata* | Puff-faced water snake | Pet trade | BGF |
| MN337848 | *Pseudoxenodon bambusicola* | Bamboo false cobra | Pet trade | BGF |
| MN337792 | *Erythrolamprus poecilogyrus* | Yellow-bellied water snake | Pet trade | BGF |
| MN337846 | *Philodryas baroni* | Baron's green racer | Pet trade | BGF |
| MN337832 | *Oxyrhopus rhombifer* | Diamondback flame snake | Pet trade | BGF |
| MN337813 | *Helicops leopardinus* | Leopard keelback | Pet trade | BGF |
| MN337842 | *Ahaetulla prasina* | Asian vine snake | Pet trade | BGF |
| MN337847 | *Platyceps florulentus* | Egyptian whip snake | Pet trade | BGF |
| MN337814 | *Thrasops jacksonii* | Black tree snake | Pet trade | BGF |
| MN337815 | *Dispholidus typus* | Boomslang | Pet trade | BGF |
| MN337811 | *Thelotornis capensis* | Savanna vine snake | Pet trade | BGF |
| MN337850 | *Trimorphodon biscutatus* | Western lyre snake | Pet trade | BGF |
| MN337810 | *Oligodon cyclurus* | Cantor's kukri snake | Pet trade | BGF |
| MT262919 | *Coelognathus radiatus* | Radiated ratsnake | Pet trade | BGF |
| JTLQ01052499 | *Pantherophis guttatus* | Corn snake | GenBank, NCBI |  |
| MN337833 | *Pantherophis spiloides* | Grey rat snake | Pet trade | FJV |
| MN337849 | *Stegonotus cucullatus* | Slaty grey snakes | Pet trade | BGF |
| MN337823 | *Dasypeltis scabra* | Common egg-eating snake | Pet trade | BGF |
| MN337793 | *Boiga irregularis* | Brown tree snake | Pet trade | BGF |
| MN337843 | *Boiga dendrophila* | Mangrove snake | Pet trade | BGF |
| XM_032237666.1 | *Thamnophis elegans* | Western terrestrial garter snake | GenBank, NCBI |  |
| MN337812 | *Natrix natrix* | European grass snake | Pet trade | FJV |
| M26389.1 | *Natrix tessellata* | Checkered water snake | GenBank, NCBI |  |
| MN337835 | *Pseudaspis cana* | Mole snake | Pet trade | BGF |
| MN337831 | *Malpolon monspessulanus* | Montpellier snake | Pet trade | BGF |
| MN337834 | *Psammophis mossambicus* | Olive grass Snake | Pet trade | BGF |
| MN337829 | *Macrelaps microlepidotus* | Natal black snake | Pet trade | BGF |
| MN337840 | *Atractaspis bibronii* | Bibron's stiletto snake | Pet trade | BGF |
| MN337839 | *Atractaspis fallax* | False mole viper | Pet trade | BGF |
| MN337821 | *Atractaspis microlepidota* | Small-scaled burrowing asp | Pet trade | BGF |
| MN337826 | *Boaedon fuliginosus* | African house snake | Pet trade | FJV |
| MN337827 | *Leioheterodon madagascariensis* | Malagasy giant hognose snake | Pet trade | BGF |
| MN337830 | *Madagascarophis ocellatus* | Ocellated cat snake | Pet trade | BGF |
| MN337805 | *Calliophis bivirgatus* | Blue Malaysian coral snake | Pet trade | BGF |
| MN337802 | *Aspidelaps lubricus* | Cape coral cobra | Pet trade | FJV |
| AF077763.1 | *Naja haje* | Egyptian cobra | GenBank, NCBI |  |
| MN337806 | *Naja kaouthia* | Monocled cobra | Pet trade | FJV |
| MN337807 | *Naja naja* | Indian cobra | National Institute of Health (NIH) Islamabad, Pakistan | MAK |
| ETE71672.1 | *Ophiophagus hannah* | King cobra | GenBank, NCBI |  |
| MN337804 | *Bungarus caeruleus* | Common krait | National Institute of Health (NIH) Islamabad, Pakistan | MAK |
| MN337816 | *Acanthophis rugosus* | Rough-scaled death adder | Gifttierhause Eimsheim, Germany. | JvT & RMW |
| XM_026696730.1 | *Pseudonaja textilis* | Eastern brown snake | GenBank, NCBI |  |
| MN337809 | *Oxyuranus microlepidotus* | Inland taipan | Pet trade | BGF |
| XM_026677744.1 | *Notechis scutatus* | Mainland tiger snake | GenBank, NCBI |  |
| MN337808 | *Hydrophis curtus* | Shaw's sea snake | Pet trade | BGF |
| MN337803 | *Aipysurus mosaicus* | Mosaic sea snake | Weipa, Queensland, Australia | BGF |
