# Supplementary material for: Widespread Evolution of Molecular Resistance to Snake Venom α-Neurotoxins in Vertebrates
Source: Toxins (Basel). 2020 Oct 2;12(10):638. doi: 10.3390/toxins12100638 (PMC7601176; doi:10.3390/toxins12100638)
Supplement: Supplementary file 1 [file toxins-12-00638-s001.zip › Supplementary File S1 Estimating LD50 values.pdf]

Estimating LD50 values

Marjolein Fokkema

Load libraries

```
library("MASS")
library("drc")
library("HelpersMG")
library("ggplot2")
library("boot")
```

Pogona

Read in data:

```
concentration <- c(0.00, 0.12, 0.24, 0.48, 7.70)
pogona_a <- c(5, 5, 5, 5, 0)
pogona_d <- 5 - pogona_a
```

Plot data (using log scale for concentration, adding a small constant to not have log of 0):

```
ggplot(data.frame(deaths = pogona_d/5, concentration = concentration + 1e-3)) +
  aes(x = concentration, y = deaths) +
  geom_point() +
  scale_x_log10()
```

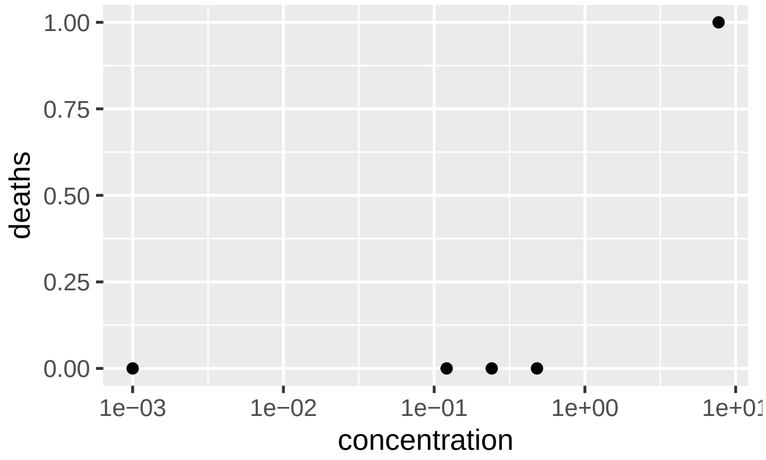

From observed data we can conclude:

- LD50 value is somewhere between 0.48 and 7.7.
- The number of levels of `concentration` and the number of samples for each level is small. The distance between concentration where no deaths, and where all deaths occur is relatively wide, so difficult to

estimate LD50 precisely.

- Estimated values for LD50 and its standard error may depend quite strongly on assumed distribution, and likely also on optimizer used for estimation.
- A model where the log of `concentration` is modelled (log-logistic model) is probably more appropriate than a probit model.

Fit a GLM probit model using maximum likelihood estimation:

```
pogona.glm <- glm(cbind(pogona_d, pogona_a) ~ concentration,
                  family = binomial(link = "probit"))
#summary(pogona.glm)
dose.p(pogona.glm, p = 0.5)
```

```
##              Dose       SE
## p = 0.5: 4.037511 5368.237
```

Plot the fitted curve:

```
newdata <- data.frame(concentration = seq(0, 8, by = .5))
newdata <- cbind(newdata, fit = predict(pogona.glm, newdata = newdata,
                                        type = "response"))
p <- ggplot(data.frame(concentration, deaths = pogona_d/5),
            aes(x=concentration, y=deaths))
p + geom_point() +
  geom_line(data=newdata, aes(y=fit)) +
  labs(x="Concentration", y="prop. deaths")
```

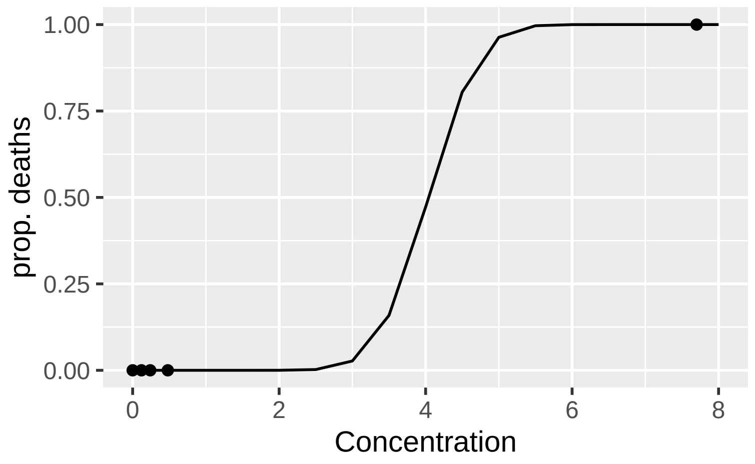

The point estimate for LD50 seems reasonable, about halfway between 0.48 and `concentration[5]`. The fitted curve fits the observed data perfectly. The standard error is huge compared to the scale of `concentration`, which does not seem reasonable. The standard errors are asymptotically correct, but with small sample size, and only a small proportion of deaths, the estimated values may be way off.

Use **drc** (Ritz et al. 2015) to fit a log-logistic model (i.e., logistic model with a parameter-dependent link function):

```
pogona.drm <- drm((pogona_d / 5) ~ concentration,
                  weights = rep(5, times = 5),
                  fct = LL.2(names = c("Slope", "LD50")), type = "binomial")
summary(pogona.drm)
```

```
##
```

```
## Model fitted: Log-logistic (ED50 as parameter) with lower limit at 0 and upper limit at 1 (2 parms)
##
## Parameter estimates:
##
##                     Estimate Std. Error t-value p-value
## Slope:(Intercept)  -14.7876  6524.6426 -0.0023  0.9982
## LD50:(Intercept)     1.8713  1123.0688  0.0017  0.9987
```

```
ED(pogona.drm, 50, interval = "delta")
```

```
##
## Estimated effective doses
##
##          Estimate Std. Error      Lower      Upper
## e:1:50     1.8713  1123.0688 -2199.3031  2203.0457
```

The LL.2 function gives the two-parameter log-logistic function:

$$f(x) = \frac{1}{(1 + \exp(b(\log(x) - \log(e))))}$$

Where x is the dose and $f(x)$ gives the predicted probability of death. We can plot the fitted model (dots are the observed datapoints):

```
plot(pogona.drm, xt = c(concentration, 1.8713), cex = .7, cex.axis=.7,
     cex.lab = .7)
```

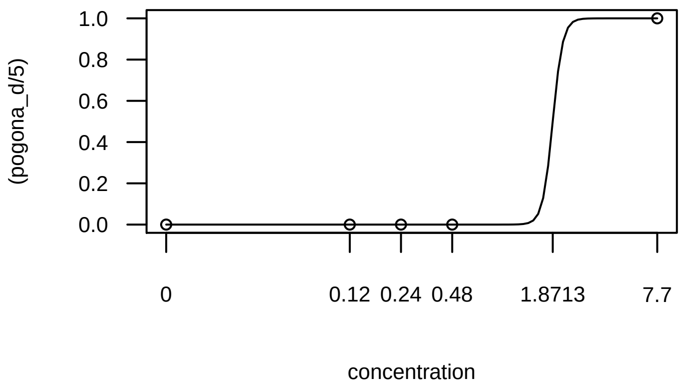

The estimated LD50 value is halfway between 0.48 and 7.7 on the log scale. The fitted curve fits the observed data perfectly. The standard error is lower than in the probit model, but still huge. Again, standard errors are asymptotically correct, but with such small sample may be way off.

- The Pogona data are quite small, deaths are only observed at the highest, somewhat extreme value of concentration. According to Faraggi (2003) “This is generally a poor design for which the maximum likelihood estimation procedure will frequently not converge”. We did not get convergence warnings, but results should be interpreted with care.
- According to Faraggi (2003), for obtaining standard errors for LD50, “the delta method cannot be recommended. Even for large sample sizes its coverage can be either strongly conservative or strongly

liberal and for small sample sizes it can provide one-tailed error rates quite far from their nominal value".

- According to Faraggi (2003), the likelihood ratio method should be preferred for computing standard errors of LD50, but it is not implemented in any **R** package currently.
- Alternatively, a saddlepoint approximation method has been proposed for computing LD50 in small samples (Paige et al. 2011), but I am not aware of any **R** implementation.
- With the current sample (small, low proportion of deaths), any standard error estimate may be unreliable. As an alternative to the ML standard errors, I used non-parametric bootstrap sampling to compute standard errors. These will underestimate true variability because the data set is very small.

```
Pogona <- data.frame(concentration = rep(concentration, each = 5),
                     death = c(rep(0, times = sum(pogona_a)),
                               rep(1, times = sum(pogona_d))))
bs.drm <- function(data, ind) {
   fit <- try(drm(death ~ concentration, data = data[ind, ],
              fct = LL.2(names = c("Slope", "LD50")), type = "binomial"))
   if (class(fit) != "try-error") ED(fit, 50, interval = "delta")[1] else NA
}
set.seed(42)
Pogona_boot <- boot(data = Pogona, statistic = bs.drm, R = 1000)
save(Pogona_boot, file = "Pogona_boot")
```

Get the standard error estimate based on bootstrap sampling:

```
load(file = "Pogona_boot")
Pogona_boot
```

```
##
## ORDINARY NONPARAMETRIC BOOTSTRAP
##
##
## Call:
## boot(data = Pogona, statistic = bs.drm, R = 1000)
##
##
## Bootstrap Statistics :
##     original      bias    std. error
## t1* 1.871903 0.04878356   0.0792652
```

We get a much smaller standard error (but we can be sure this underestimates true variability because of the small sample size).

Get intervals:

```
boot.ci(Pogona_boot)
```

```
## Warning in boot.ci(Pogona_boot): bootstrap variances needed for studentized
## intervals

## Warning in norm.inter(t, adj.alpha): extreme order statistics used as endpoints

## BOOTSTRAP CONFIDENCE INTERVAL CALCULATIONS
## Based on 998 bootstrap replicates
##
## CALL :
## boot.ci(boot.out = Pogona_boot)
##
```

```
## Intervals :
## Level      Normal              Basic
## 95%   ( 1.668,  1.978 )   ( 1.693,  1.968 )
##
## Level     Percentile            BCa
## 95%   ( 1.776,  2.051 )   ( 1.276,  1.973 )
## Calculations and Intervals on Original Scale
## Warning : BCa Intervals used Extreme Quantiles
## Some BCa intervals may be unstable
```

Chicken

Read in data:

```
chicken_a <- c(5, 5, 5, 0, 0)
chicken_d <- 5 - chicken_a
```

Plot data:

```
ggplot(data.frame(deaths = chicken_d/5, concentration = concentration + 1e-3)) +
  aes(x = concentration, y = deaths) +
  geom_point() +
  scale_x_log10()
```

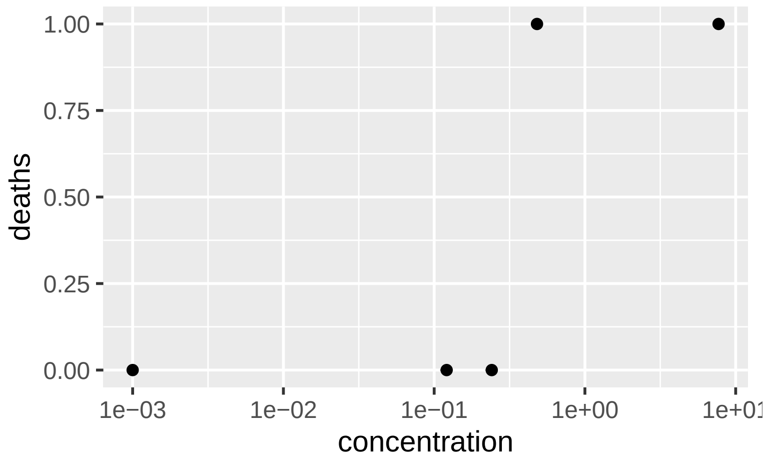

The LD50 value will be somewhere between 0.24 and 0.48. The exact point estimate and standard error will depend on the assumed distribution.

Fit a probit regression using ML estimation:

```
chicken.glm <- glm(cbind(chicken_d, chicken_a) ~ concentration,
                   family = binomial(link = "probit"))
```

```
## Warning: glm.fit: algorithm did not converge

## Warning: glm.fit: fitted probabilities numerically 0 or 1 occurred
```

```
#summary(chicken.glm)
dose.p(chicken.glm, p = 0.5)
```

```
##               Dose       SE
## p = 0.5: 0.3588565 46.97016
```

Warning is not worrysome for point estimates, but estimated standard errors may be unreliable. Standard error is much lower than with Pogona data, but still quite large.

Fit a log-logistic model:

```
chicken.drm <- drm((chicken_d / 5) ~ concentration, weights = rep(5, times = 5),
                  fct = LL.2(names = c("Slope", "LD50")), type = "binomial")
#summary(stickleback.drm)
ED(chicken.drm, 50, interval = "delta")
```

```
##
## Estimated effective doses
```

```
##
##        Estimate Std. Error    Lower    Upper
## e:1:50  0.33975    1.36638 -2.33830  3.01781
```

Get quite similar LD50 value, but standard error for log-logistic model much lower (and seems more reasonable) than for probit model.

Perform bootstrap sampling to obtain standard error:

```
Chicken <- data.frame(concentration = rep(concentration, each = 5),
                      death = c(rep(0, times = sum(chicken_a)),
                                rep(1, times = sum(chicken_d))))
set.seed(42)
Chicken_boot <- boot(data = Chicken, statistic = bs.drm, R = 1000)
save(Chicken_boot, file = "Chicken_boot")
```

```
load(file = "Chicken_boot")
Chicken_boot
```

```
##
## ORDINARY NONPARAMETRIC BOOTSTRAP
##
##
## Call:
## boot(data = Chicken, statistic = bs.drm, R = 1000)
##
##
## Bootstrap Statistics :
##     original       bias    std. error
## t1* 0.339754 0.004008534  0.07397171
```

```
boot.ci(Chicken_boot)
```

```
## Warning in boot.ci(Chicken_boot): bootstrap variances needed for studentized
## intervals

## BOOTSTRAP CONFIDENCE INTERVAL CALCULATIONS
## Based on 1000 bootstrap replicates
##
## CALL :
## boot.ci(boot.out = Chicken_boot)
##
## Intervals :
## Level      Normal              Basic
## 95%   ( 0.1908,  0.4807 )   ( 0.3324,  0.3495 )
##
## Level     Percentile            BCa
## 95%   ( 0.3300,  0.3471 )   ( 0.3326,  0.3488 )
## Calculations and Intervals on Original Scale
```

With bootstrap sampling, get much lower standard error.

Stickleback

Read in data:

```
concentration2 <- c(0, 0.03, 0.06, 0.12, 0.24, 0.48, 0.945, 1.89, 3.78, 7.7)
stickleback_a <- c(8, 8, 8, 8, 8, 8, 0, 0, 0, 0)
stickleback_d <- 8 - stickleback_a
```

Plot data:

```
ggplot(data.frame(deaths = stickleback_d/8, concentration = concentration2 + 1e-3)) +
  aes(x = concentration, y = deaths) +
  geom_point() +
  scale_x_log10()
```

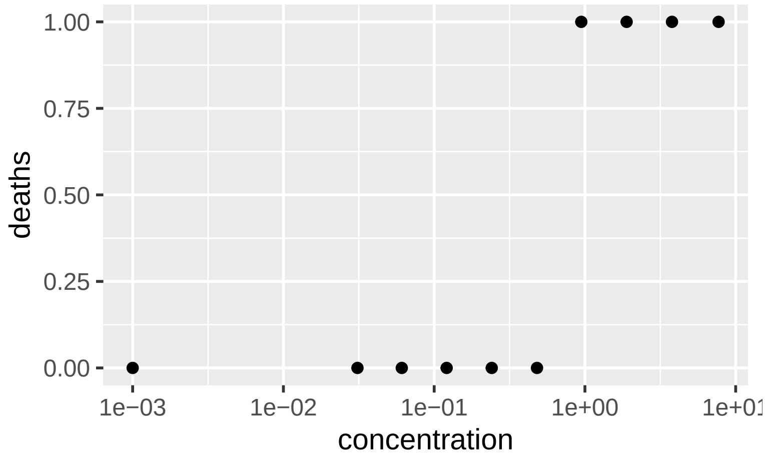

The LD50 value will be somewhere between 0.48 and 0.945. The exact point estimate and standard error will depend on the assumed distribution.

Fit a probit resgression using ML estimation:

```
stickleback.glm <- glm(cbind(stickleback_d, stickleback_a) ~ concentration2,
                       family = binomial(link = "probit"))
```

```
## Warning: glm.fit: algorithm did not converge
```

```
## Warning: glm.fit: fitted probabilities numerically 0 or 1 occurred
```

```
#summary(stickleback.glm)
dose.p(stickleback.glm, p = 0.5)
```

```
##                Dose       SE
## p = 0.5: 0.7113159 58.26822
```

Fit a log-logistic model:

```
stickleback.drm <- drm((stickleback_d / 8) ~ concentration2, weights = rep(8, times = 10),
                  fct = LL.2(names = c("Slope", "LD50")), type = "binomial")
#summary(stickleback.drm)
ED(stickleback.drm, 50, interval = "delta")
```

```
##
## Estimated effective doses
##
##          Estimate Std. Error    Lower    Upper
```

```
## e:1:50  0.67189    2.99228 -5.19287  6.53666
```

Again, much lower standard error for log-logistic than for probit model.

Compute bootstrap confidence interval:

```r
Stickleback <- data.frame(concentration = rep(concentration2, each = 8),
                     death = c(rep(0, times = sum(stickleback_a)),
                               rep(1, times = sum(stickleback_d))))
set.seed(42)
Stickleback_boot <- boot(data = Stickleback, statistic = bs.drm, R = 1000)
save(Stickleback_boot, file = "Stickleback_boot")
```

```r
load(file = "Stickleback_boot")
Stickleback_boot
```

```
##
## ORDINARY NONPARAMETRIC BOOTSTRAP
##
##
## Call:
## boot(data = Stickleback, statistic = bs.drm, R = 1000)
##
##
## Bootstrap Statistics :
##      original      bias    std. error
## t1* 0.6718933 0.00119255  0.01005046
```

```r
boot.ci(Stickleback_boot)
```

```
## Warning in boot.ci(Stickleback_boot): bootstrap variances needed for studentized
## intervals

## BOOTSTRAP CONFIDENCE INTERVAL CALCULATIONS
## Based on 1000 bootstrap replicates
##
## CALL :
## boot.ci(boot.out = Stickleback_boot)
##
## Intervals :
## Level      Normal              Basic
## 95%   ( 0.6510,  0.6904 )   ( 0.6604,  0.6804 )
##
## Level     Percentile            BCa
## 95%   ( 0.6634,  0.6834 )   ( 0.6592,  0.6811 )
## Calculations and Intervals on Original Scale
```

Standard error based on bootstrap sampling is much lower than the ML standard errors (but likely underestimates true variability).

Zebrafish

Read in data:

```
zebrafish_a <- c(8, 8, 5, 0, 0, 0, 0, 0, 0, 0)
zebrafish_d <- 8 - zebrafish_a
```

Plot data:

```
ggplot(data.frame(deaths = zebrafish_d/8, concentration = concentration2 + 1e-3)) +
  aes(x = concentration, y = deaths) +
  geom_point() +
  scale_x_log10()
```

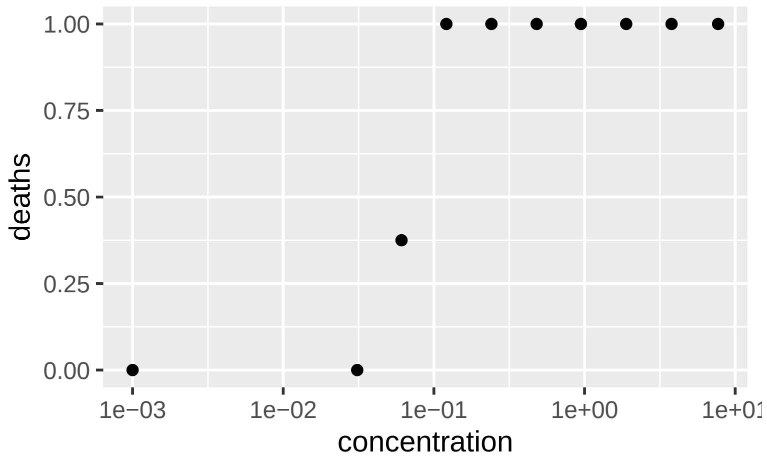

The LD50 value will be somewhere between 0.24 and 0.48. The exact point estimate and standard error will depend on the assumed distribution.

Fit a probit regression lusing ML:

```
zebrafish.glm <- glm(cbind(zebrafish_d, zebrafish_a) ~ concentration2,
                     family = binomial(link = "probit"))
```

```
## Warning: glm.fit: algorithm did not converge

## Warning: glm.fit: fitted probabilities numerically 0 or 1 occurred
```

```
#summary(zebrafish.glm)
dose.p(zebrafish.glm, p = 0.5)
```

```
##               Dose       SE
## p = 0.5: 0.061827 0.112384
```

Fit a log-logistic model:

```
zebrafish.drm <- drm((zebrafish_d / 8) ~ concentration2, weights = rep(8, times = 10),
                  fct = LL.2(names = c("Slope", "LD50")), type = "binomial")
#summary(zebrafish.drm)
ED(zebrafish.drm, 50, interval = "delta")
```

```
##
## Estimated effective doses
##
##          Estimate Std. Error     Lower     Upper
## e:1:50 0.0620646  0.0091304 0.0441694 0.0799598
```

standard errors for both probit and log-logistic model are small, but much smaller for log-logistic model.

```
Zebrafish <- data.frame(concentration = rep(concentration2, each = 8),
                     death = c(rep(1, times = sum(zebrafish_a)),
                               rep(0, times = sum(zebrafish_d))))
set.seed(42)
Zebrafish_boot <- boot(data = Zebrafish, statistic = bs.drm, R = 1000)
save(Zebrafish_boot, file = "Zebrafish_boot")
```

```
load(file = "Zebrafish_boot")
Zebrafish_boot
```

```
##
## ORDINARY NONPARAMETRIC BOOTSTRAP
##
##
## Call:
## boot(data = Zebrafish, statistic = bs.drm, R = 1000)
##
##
## Bootstrap Statistics :
##       original        bias    std. error
## t1* 0.06205744 0.0008356149 0.005331227
```

```
boot.ci(Zebrafish_boot)
```

```
## Warning in boot.ci(Zebrafish_boot): bootstrap variances needed for studentized
## intervals

## BOOTSTRAP CONFIDENCE INTERVAL CALCULATIONS
## Based on 1000 bootstrap replicates
##
## CALL :
## boot.ci(boot.out = Zebrafish_boot)
##
## Intervals :
## Level      Normal              Basic
## 95%   ( 0.0508,  0.0717 )   ( 0.0402,  0.0676 )
##
## Level     Percentile            BCa
## 95%   ( 0.0565,  0.0839 )   ( 0.0555,  0.0679 )
## Calculations and Intervals on Original Scale
```

The bootstrap-based standard error is somewhat smaller than the ML-based standard error.

References

Faraggi, D., Izikson, P., & Reiser, B. (2003). Confidence intervals for the 50 percent response dose. Statistics in Medicine, 22(12), 1977-1988.

Paige, R. L., Chapman, P. L., & Butler, R. W. (2011). Small sample LD50 confidence intervals using saddlepoint approximations. Journal of the American Statistical Association, 106(493), 334-344.

Ritz, C., Baty, F., Streibig, J. C., & Gerhard, D. (2015). Dose-response analysis using R. PloS one, 10(12).
