# Supplementary material for: Widespread Evolution of Molecular Resistance to Snake Venom α-Neurotoxins in Vertebrates
Source: Toxins (Basel). 2020 Oct 2;12(10):638. doi: 10.3390/toxins12100638 (PMC7601176; doi:10.3390/toxins12100638)
Supplement: Supplementary file 1 [file toxins-12-00638-s001.zip › Supplementary File S2 Primers used in the current study for amplification of Chrna1 (cholinergic receptor nicotinic alpha 1 subunit) ligand binding site..docx]

**Supplementary File S2:** Primers used in the current study for amplification of Chrna1 (cholinergic receptor nicotinic alpha 1 subunit) ligand binding site.

| **Primers used for snakes** | |
| --- | --- |
|  |  |
| F1 | TGGAAGCATTTTCCTTTTCAGGAA |
| F2 | AGAATGGGCAATGAAAGATTACC |
| R2 | GAATGAGAAGAGAAGGCAAGGAAT |
| R1 | AGTCAAGAATGAGAAGAGAAGGCA |
|  |  |
|  | Added to the 5'end was an M13 tail as follows: |
| M13F | TGTAAAACGACGGCCAGT |
| M13R | CAGGAAACAGCTATGAC |
|  |  |
| **Primers used for birds** | |
|  |  |
| F1 | CCTGATCTGAGTAACTTCATGGAGAG |
| F2 | TTTCTGTCAGGAGAGCGATCG |
| R1 | AAGGAGAAGAGCAGGCAGGG |
| R2 | TAAATTTACATACCTGAATCTGTGGG |
|  | Added to the 5'end was an M13 tail as follows: |
|  | GTTTTCCCAGTCACGAC |
|  | CAGGAAACAGCTATGAC |
|  |  |
| **Primers used for lizards *Anolis*, *Pogona* and *Ophisaurus gracilis*** | |
| F1 | TAGGTAAGTGAACGTCCAGAC |
| F2 | TCCAGACCTGAGTAACTACATGG |
| F3 (alternative) | TGAGTAACTACATGGGGAGTGG |
| R1 | TGTGGGTAGATAAAACACTAATCC |
| R2 | AATGAGAACAGGAGGCAAGG |
|  |  |
|  | Added to the 5'end was an M13 tail as follows: |
|  | TGTAAAACGACGGCCAGT |
|  | CAGGAAACAGCTATGAC |
