# Supplementary material for: Widespread Evolution of Molecular Resistance to Snake Venom α-Neurotoxins in Vertebrates
Source: Toxins (Basel). 2020 Oct 2;12(10):638. doi: 10.3390/toxins12100638 (PMC7601176; doi:10.3390/toxins12100638)
Supplement: Supplementary file 1 [file toxins-12-00638-s001.zip › Supplementary Note S1. Preliminary (independent) analysis of a subset of the data..docx]

Supplementary Note 1. Preliminary (independent) analysis of a subset of the data.

Our aim here was to make a preliminary determination of potential sites under positive selection. It was carried out independently of the other analysis by one of us (DK) on a smaller subset of the main sequence set. It utilised the Selecton server and EasyCodeML progamme (M8-M8a, Bayes Empirical Bayes (BEB)), along with the Datamonkey server (Branch-site Unrestricted Statistical Test for Episodic Diversification (BUSTED), Single Likelihood Ancestral Counting (SLAC), Fixed Effects Likelihood (FEL), MEME and FUBAR approaches).

The datasets used to infer the action of site-specific positive selection in snake nAChR genes were different: a) in the loop C of the nAChR we analysed 41 sequences with 42 codons (15 Cbtx resistant snake species, 4 non-resistant snake species, 15 non-resistant vertebrates and 7 snake venom resistant mammals) and b) the whole nAChR was analysed separately in reptiles, mammals or in all species combined. Species for the two analyses are listed in Box A. We analysed our gene sets using Selecton [1,2], EasyCodeML [3]and Datamonkey [4] to infer site-specific selection pressures acting on snake nAChR genes, and to compare the results with known structural and experimental data.

Evolution of snake nAChR genes was first evaluated under several standard models of sequence evolution as implemented in the Selecton. We used the site-specific model that accounts for the rate variation across the sites. Estimates using the BEB approach implemented in M8 model suggested that in Cbtx resistant snake nAChRs nine codons are under positive selection—each of these sites had a posterior expectation of ω higher than 1 with a posterior probability (PP) of at least 0.95. Six positively selected sites have PP ≥ 0.95 (corresponding to codon positions 172, 177, 181, 187, 194 and 206) and 3 sites have PP ≥ 0.99 (corresponding to codon positions 189, 191 and 195; Box B1). With the EasyCodeML we found three positively selected sites corresponding to codon positions 187, 189 and 194 (Box B2).

Positive selection, as detected by the Selecton and EasyCodeML, has been conclusively supported by the Mixed Effects Model of Evolution (MEME), Branch-site Unrestricted Statistical Test for Episodic Diversification (BUSTED), Fast Unconstrained Bayesian AppRoximation (FUBAR) [5], Fixed Effects Likelihood (FEL) and Single Likelihood Ancestral Counting (SLAC) [6] implemented through the Datamonkey server [4]. MEME, BUSTED, FUBAR, FEL and SLAC have identified a different set of positively selected codons (Box C1). The MEME and BUSTED models found the largest number of positively selected codons (five), followed by the FUBAR (four), SLAC (only one), and FEL (only one); Box C1. Some sites were identified only by the Selecton models or by the Datamonkey models (Boxes B and C). Evidence provided by various analyses (M8, MEME, BUSTED, FUBAR, FEL and SLAC) revealed the action of positive diversifying selection on the snake venom nAChR genes.

We compared the amount of positively selected sites in snake nAChRs with two previous studies that also included analysis of positively selected sites in nAChRs, one in venom resistant mammals [7] and the other in snakes [8]. No positively selected sites were found in snake nAChRs; however, they were found in three finger toxins that interact with the nAChRs [8]. Under M8 model only two positively selected sites were found in venom resistant mammals. From our analysis it is evident that snake venom nAChRs possess the highest number of positively selected sites in the nAChR family.

By using diverse models we demonstrated the presence of positively selected sites in the loop C of nAChRs. Although the Selecton, EasyCodeML and Datamonkey models slightly differ in the numbers of inferred positively selected sites, the majority of them are located in structurally and functionally important regions of the nAChR proteins, especially in the loop C which is the major component of the principal face of the subunit interface (ligand binding site) [9].

However, the analysis of positive selection in the whole nAChR genes with MEME provided quite unexpected results. While in mammals resistant to snake venom, only two positively selected sites (PSS) were found, in the combined reptiles and mammals dataset we found five PSS. Yet, only in the reptiles analysed, did the MEME model fid evidence of episodic positive selection at 12 sites (codon positions) with p-value threshold of 0.1. (Box C2). Four PSS are located in the loop C (codons 178, 181, 189 and 194) while seven PSS are located at the 5’ end of the nAChR gene and only a single PSS is located at the 3’ end of the gene. It is important that 11 PSS are located in the extracellular domain of the nAChR (LGIC_ECD domain). Positively selected sites in this domain of the snake nAChRs are located in α1 helix (2 PSS), loop 1 (3 PSS), loop between β2 and β3 strands (1 PSS), β6 strand (1 PSS), β9 strand (3 PSS; it overlaps partially with the loop C, where in the tip of the loop C two PSS are located). The locations of the PSS in mammals are different, one is in the β5’ strand and one is in the tip of the loop C. These additional snake-specific PSS may be located at the nAChR dimer interfaces or in the ligand binding pocket [9].

Methods

As a preliminary screen of sites under positive selection was performed by one of us (DK) on a smaller subset of the main sequence collection. The Selecton server (Server for the Identification of Site-Specific Positive and Purifying Selection, version 2.4; http://selecton.tau.ac.il/index.html) has been used to identify the ratio of nonsynonymous and synonymous substitutions (dN/dS; termed ω) at each codon site based on an empirical Bayesian method. The pressure of selection can induce either purifying or positive selection at specific areas of the sequence where sites with ω values significantly higher or lower than one are an indication of positive or purifying selection, respectively [10]. We analysed 41 aligned coding sequences of the ligand-binding domain of snake nAChR. To identify codons under positive selection, we used M8 vs. M8a test. M8 assumes a beta distribution, from 0 to 1, of ω for sites and an additional class of sites under positive selection (ω > 1), while M8a acts as a null model by fixing this last class of sites at ω = 1. Following these analyses, a likelihood ratio test was conducted to determine if there were significant likelihood gains by allowing positive selection. The results from Selecton version 2.4 are visualised with seven-color scale for representing the different types of selection. The Bayes empirical Bayes (BEB) approach [11] was used to identify amino acids under positive selection by calculating the posterior probabilities that a particular amino acid belongs to a given selection class (neutral, conserved or highly variable). Sites with greater posterior probability (PP ≥ 95%) of belonging to the ‘ω > 1 class’ were inferred to be positively selected. We also used CodeML program in EasyCodeML package [3].

The impact of positive selection was further assessed using additional codon models to estimate the rates of synonymous and nonsynonymous substitutions [6]. Five methods implemented on the DATAMONKEY web server [4] (http://www.datamonkey.org/), the Mixed Effects Model of Evolution (MEME) [12], BUSTED [13], Single Likelihood Ancestral Counting (SLAC) [6], Fixed Effects Likelihood (FEL), and FUBAR [5], were used. We used the same dataset as in the Selecton and EasyCodeML analyses as well as the whole nAChR that was analysed separately in snakes, mammals or in all species combined. After reconstructing ancestral sequences, SLAC compares normalised expected and observed numbers of synonymous and nonsynonymous substitutions per variable site. FEL compares the instantaneous synonymous site rate (α) and the instantaneous nonsynonymous site rate (β) on a per site basis, without assuming a prior dN/dS distribution. Sites with cut-off values of *p* < 0.1in SLAC and FEL and PP < 0.9 in FUBAR were considered as candidates to have evolved under positive selection. In all analyses performed in DATAMONKEY, the most suited model of evolution for each data set, directly estimated on this web server, was used. In addition, the mixed effects model of evolution (MEME), a branch-site method incorporated in the DATAMONKEY server, was used to test for both pervasive and episodic diversifying selection [12]. MEME is a generalization of FEL but models variable ω across lineages at individual sites, restricting ω to be ≤1 in a proportion p of branches and unrestricted at a proportion (1—p) of branches per site. Positive selection was inferred with this method for a *p* value < 0.1.

Box A. Species analysed.

| **1) loop C of the nAChR**  *Calliophis bivirgatus*  *Anilios bituberculatus*  *Boa constrictor*  *Vombatus ursinus*  *Grammomys surdaster*  *Mellivora capensis*  *Suricata suricatta*  *Otolemur garnettii*  *Sorex araneus*  *Chrysochloris asiatica*  *Orycteropus afer*  *Sus scrofa*  *Erinaceus europaeus*  *Homo sapiens*  *Pongo abelii*  *Gallus gallus*  *Sphenodon punctatus*  *Rhinatrema bivittatum* | *Microcaecilia unicolor*  *Chelonia mydas*  *Terrapene carolina*  *Gopherus evgoodei*  *Crocodylus porosus*  *Alligator mississippiensis*  *Pogona vitticeps*  *Helicops leopardinus*  *Atractaspis microlepidota*  *Coelegnathus radiatus*  *Dispholidus typus*  *Thelotornis capensis*  *Thrasops jacksonii*  *Natrix natrix*  *Aipysurus mosaicus*  *Pseudonaja textilis*  *Acanthophis rugosus*  *Notechis scutatus*  *Bungarus caeruleus* | *Aspidelaps lubricus infuscates*  *Natrix tessellata*  *Naja kaouthia*  *Naja haje*  **2) whole nAChR**  *Pogona vitticeps*  *Protobothrops mucrosquamatus*  *Pseudonaja textilis*  *Notechis scutatus*  *Crocodylus porosus*  *Chelonia mydas*  *Terrapene carolina*  *Vombatus ursinus*  *Erinaceus europaeus*  *Suricata suricatta*  *Homo sapiens*  *Sus scrofa* |
| --- | --- | --- |

Box B. Positively selected sites under M8 site model.

**1) Analysis of the nAChR loop C (42 codons, 41 species) by Selecton**


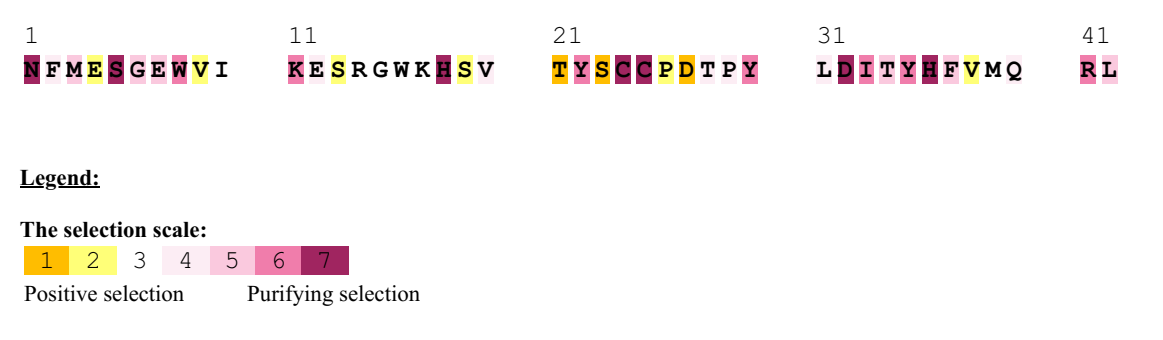


Positively selected sites inferred under posterior probability PP > 0.95

Codons: 4, 9, 13, 19, 26 and 38

Amino acids in mature protein: 172, 177, 181, 187, 194 and 206.

Positively selected sites inferred under posterior probability PP > 0.99

Codons: 21, 23 and 27

Amino acids in mature protein: 189, 191 and 195.

**2) Analysis of the nAChR loop C (42 codons, 41 species) by EasyCodeML**

Positively selected sites inferred

Codons: 19, 21, and 26

Amino acids in mature protein: 187, 189 and 194.

Supplementary References

Box C. Positively selected sites under MEME, BUSTED, FUBAR, FEL and SLAC models.

**1) Analysis of the nAChR loop C (42 codons, 41 species)**

| evolutionary model | number of positively selected sites | *p* value/posterior probability | codons | amino acids |
| --- | --- | --- | --- | --- |
| MEME | 5 | *p* val = 0.1 | 17, 21, 23, 26, 27 | 185, 189, 191, 194, 195 |
| BUSTED | 5 | *p* val = 0.1 | 13, 21, 23, 26, 27 | 181, 189, 191, 194, 195 |
| FUBAR | 4 | PP = 0.9 | 13, 21, 23, 27 | 181, 189, 191, 195 |
| FEL | 1 | *p* val = 0.1 | 23 | 191 |
| SLAC | 1 | *p* val = 0.1 | 23 | 191 |

**2) Analysis of the whole nAChR mRNAs (including signal peptide (SP)) under the MEME model (*p* val = 0.1) (468 codons).** Signal peptide is 31 amino acids long in snakes and 20 in mammals.

| sequences analysed/number of species | Number of positively selected sites | codons | amino acids in the mature protein |
| --- | --- | --- | --- |
| reptiles only/7 | 15 (3 in SP) | 15, 19, 26, 34, 45, 48, 54, 57, 101, 148, 209, 212, 220, 225, 427 | 3, 14, 17, 23, 26, 70, 117, 178, 181, 189, 194, 396 |
| reptiles and mammals/12 | 9 (4 in SP) | 14, 18, 19, 25, 115, 116, 225, 239, 437 | 84, 85, 194, 208, 406 |
| mammals only/5 | 2 | 131, 215 | 111, 195 |

1. Doron-Faigenboim, A.; Stern, A.; Mayrose, I.; Bacharach, E.; Pupko, T. Selecton: a server for detecting evolutionary forces at a single amino-acid site. *Bioinformatics* **2005**, *21*, 2101–2103, doi:10.1093/bioinformatics/bti259.
2. Stern, A.; Doron-Faigenboim, A.; Erez, E.; Martz, E.; Bacharach, E.; Pupko, T. Selecton 2007: advanced models for detecting positive and purifying selection using a Bayesian inference approach. *Nucleic Acids Res.* **2007**, *35*, W506–511, doi:10.1093/nar/gkm382.
3. Gao, F.; Chen, C.; Arab, D.A.; Du, Z.; He, Y.; Ho, S.Y.W. EasyCodeML: A visual tool for analysis of selection using CodeML. *Ecol. Evol.* **2019**, *9*, 3891–3898, doi:10.1002/ece3.5015.
4. Weaver, S.; Shank, S.D.; Spielman, S.J.; Li, M.; Muse, S.V.; Kosakovsky Pond, S.L. Datamonkey 2.0: A Modern Web Application for Characterizing Selective and Other Evolutionary Processes. *Mol. Biol. Evol.* **2018**, *35*, 773–777, doi:10.1093/molbev/msx335.
5. Murrell, B.; Moola, S.; Mabona, A.; Weighill, T.; Sheward, D.; Pond, K.; L, S.; Scheffler, K. FUBAR: A Fast, Unconstrained Bayesian AppRoximation for Inferring Selection. *Mol. Biol. Evol.* **2013**, *30*, 1196–1205, doi:10.1093/molbev/mst030.
6. Kosakovsky Pond, S.L.; Frost, S.D. Not so different after all: a comparison of methods for detecting amino acid sites under selection. *Mol. Biol. Evol.* **2005**, *22*, 1208–1222, doi:10.1093/molbev/msi105.
7. Drabeck, D.H.; Dean, A.M.; Jansa, S.A. Why the honey badger don't care: Convergent evolution of venom-targeted nicotinic acetylcholine receptors in mammals that survive venomous snake bites. *Toxicon* **2015**, *99*, 68–72, doi:10.1016/j.toxicon.2015.03.007.
8. Ji, X.H.; Zhang, S.F.; Gao, B.; Zhu, S.Y. Receptor variability-driven evolution of snake toxins. *Zool. Res.* **2018**, *39*, 431–436, doi:10.24272/j.issn.2095-8137.2018.063.
9. Brejc, K.; van Dijk, W.J.; Klaassen, R.V.; Schuurmans, M.; van Der Oost, J.; Smit, A.B.; Sixma, T.K. Crystal structure of an ACh-binding protein reveals the ligand-binding domain of nicotinic receptors. *Nature* **2001**, *411*, 269–276, doi:10.1038/35077011.
10. Yang, Z.; Nielsen, R. Codon-substitution models for detecting molecular adaptation at individual sites along specific lineages. *Mol. Biol. Evol.* **2002**, *19*, 908–917, doi:10.1093/oxfordjournals.molbev.a004148.
11. Yang, Z.; Wong, W.S.; Nielsen, R. Bayes empirical bayes inference of amino acid sites under positive selection. *Mol. Biol. Evol.* **2005**, *22*, 1107–1118, doi:10.1093/molbev/msi097.
12. Murrell, B.; Wertheim, J.O.; Moola, S.; Weighill, T.; Scheffler, K.; Pond, S.L.K. Detecting Individual Sites Subject to Episodic Diversifying Selection. *PLOS Genetics* **2012**, *8*, e1002764, doi:10.1371/journal.pgen.1002764.
13. Murrell, B.; Weaver, S.; Smith, M.D.; Wertheim, J.O.; Murrell, S.; Aylward, A.; Eren, K.; Pollner, T.; Martin, D.P.; Smith, D.M., et al. Gene-wide identification of episodic selection. *Mol. Biol. Evol.* **2015**, *32*, 1365–1371, doi:10.1093/molbev/msv035.
